# A haplotype-phased genome characterizes the genomic architecture and causal variants for *RXf1* conferring resistance to *Xanthomonas fragariae* in strawberry (*F*. × *ananassa*)

**DOI:** 10.1186/s12864-025-11517-w

**Published:** 2025-05-08

**Authors:** Jin-Hee Kim, Vance M. Whitaker, Seonghee Lee

**Affiliations:** 1 Gulf Coast Research and Education Center, Institute of Food and Agriculture Science, University of Florida, Wimauma, FL 33598 USA; 2 Horticultural Science Department, University of Florida, Gainesville, FL 32611 USA

**Keywords:** Polyploid, Octoploid, Bacterial angular leaf spot (ALS), Disease resistance, Fine-mapping, RNA-sequencing, Whole genome assembly

## Abstract

**Background:**

Cultivated octoploid strawberry (*Fragaria* × *ananassa*) is one of the most economically important fruits worldwide due to its flavor, texture, and health benefits. However, bacterial angular leaf spot (ALS) causes economic losses in fruit production and plant nurseries. All commercial strawberry varieties are susceptible to ALS. A major resistance locus, *RXf1*, has been reported, but the genomic structure and candidate genes underlying this resistance remain known.

**Results:**

Fine-mapping was performed using three segregating populations containing 663 individuals that were genotyped with subgenome specific seven high-resolution melting (HRM) markers to narrow the *RXf1* region to a 486-kb interval on chromosome 6C. We assembled a haplotype-phased chromosome-scale genome of ALS-resistant breeding selection FL17.68–110 using highly accurate long-read sequencing and trio-binning with parental short reads. The 1.62 Gbp genome containing two haplotypes, 56 chromosomes and 193,072 annotated genes. Transcriptome analysis in response to the ALS pathogen identified a candidate gene, *Resistance gene analogue 3* (*RGA3*), associated with the *RXf1* resistance. The gene structure and sequence variations within *FaRGA3* were identified between resistant and susceptible genotypes.

**Conclusions:**

Our results narrowed the *RXf1* region, identified structural variations within this locus and pointed to *FaRGA3* as a promising candidate gene. This information will be useful for breeders toward developing ALS-resistant strawberry varieties, and the high-quality genome will be a valuable resource for further genomics research in octoploid strawberry.

**Supplementary Information:**

The online version contains supplementary material available at 10.1186/s12864-025-11517-w.

## Introduction

The octoploid cultivated strawberry (*Fragaria* × *ananassa* Duch.; 2n = 8 × = 56) is one of the most consumed and economically important fruits in the world. Over 9.2 million tons of fresh strawberries were produced in 2021 [1, 2]. In the United States, strawberry production was 1.2 million tons valued at $3.4 billion in 2021, making the US the second largest producer [1, 2]. Numerous diseases afflict economic losses in strawberry production, making resistance traits a high priority in breeding [3, 4]. Breeding is a highly effective method of control, avoiding problems of chemical controls such as the development of resistance to pesticides in pathogens, application costs, and sustainability concerns [5].

Angular leaf spot (ALS) is a bacterial disease of strawberry caused by *Xanthomonas fragariae*, with symptoms appearing on strawberry leaves, sepals, and crowns [6–8]. ALS in cultivated strawberries was first reported in Minnesota, United States in 1960, and has since been reported on other continents, including South America, Europe, Africa, Oceania, and Asia. *X. fragariae* is considered a quarantine organism by the European and Mediterranean Plant Protection Organization (EPPO) and Interafrican Phytosanitary Council (IAPSC) [9]. In Wisconsin, ALS was reported to cause 70% to 80% strawberry yield loss [10, 11]. Studies of infected fields in Florida showed an approximately 8% reduction in fruit production, representing significant economic loss to growers [12]. Consequently, the development of ALS-resistant strawberry varieties would be highly desirable for both fruit production and plant production in nurseries.

Developing ALS-resistant varieties with elite traits is challenging because the best ALS resistance sources for breeding of cultivated strawberries are from the wild octoploid species *F. virginiana* [13]. Resistance sources were screened in different ploidy levels of strawberry plants and found in diploid [6, 7], tetraploid, hexaploid, octoploid, and decaploid strawberries [14, 15]. Two wild octoploid strawberry genotypes, US 4808 (SG-89; *F. virginiana*) and US 4809 (80–4–38; Crossed between *F. virginiana* clone SG-26 and *F.* × *ananassa* ‘Earliglow’), were reported as ALS-resistant [14]. Jamieson et al. conducted backcrossing of these accessions with *F.* × *ananassa* and phenotyped the progenies [13]. Although the resistant plants exhibit undesirable traits liked to the ALS resistance, such as non-marketable fruit, variegation, pistillate flowers, and susceptibility to other pathogens, these drawbacks could be mitigated by breaking the genetic linkage through increasing population size and the number of backcrossing generations [13]. This backcrossing scheme was continued in the University of Florida strawberry breeding program where four full-sib families were developed for ALS resistance and validated for the genetic architecture of the resistance [16]. Quantitative trait loci (QTL) analysis was performed using the IStraw90 Axiom® single nucleotide polymorphisms (SNPs) array, and a major locus for ALS resistance was identified and defined as *RXf1* (*FaRXf1*) [16, 17]. *RXf1* was located to linkage group 6D (LG6D) and to the interval 32.74 Mbp – 33.66 Mbp in the *Fragaria vesca* genome for both resources [16]. Once high-quality octoploid strawberry reference genomes and the new *F.* × *ananassa*-based 50 K FanaSNP array became available, the location of *RXf1* was determined at chromosome 6–2 in the ‘Camarosa’ genomes [18, 19]. Additionally, subgenome-specific markers for ALS resistance selection were developed and applied for marker-assisted seedling selection (MASS) [20]. We do not yet know the genomic architecture and genes associated with the resistance at *RXf1*. This may be largely due to the fact that all genomes available until now are from individuals susceptible to ALS.

In this study, we constructed a chromosome-scale haplotype-phased genome of the ALS resistant variety (FL17.68–110) and conducted a comprehensive characterization of the genomic structure of *RXf1* with multiple reference genomes of octoploid strawberry. Furthermore, transcriptomic and comparative genomic analyses identified a candidate gene, *FaRGA3*, with strong evidence of association with *RXf1*-mediated resistance to ALS.

## Results

### Fine-mapping of *RXf1* conferring resistance to ALS

**Fig. 1:**
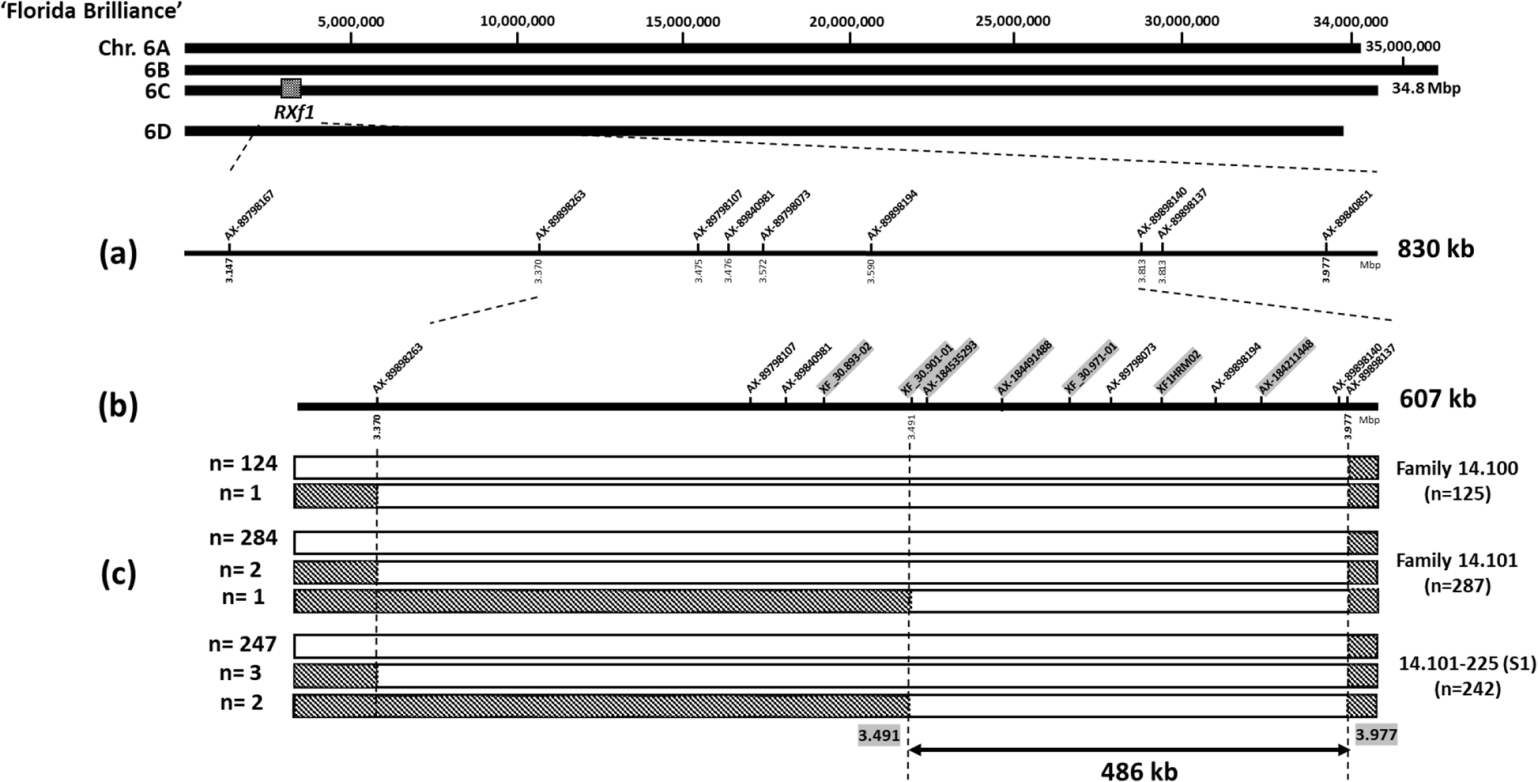
Fine mapping of *RXf1*. **a** *RXf1*, a major QTL conferring ALS resistance in the octoploid strawberry, was located between marker ‘AX-89798167’ and ‘AX-89840851’ on chromosome 6Ca in the octoploid strawberry ‘Florida Brilliance’ genome (FaFB1). **b** Seven newly developed markers were mapped between previously developed markers. The new markers were highlighted in grey. **c** Eight recombinants except one overlapped accession were identified at 3.370 or 3.491 Mbp from the left side of *RXf1*, and none of the recombinants was found at the right side of *RXf1*. *RXf1* was narrowed to the 486-kb interval region between marker XF_30.901–01 and AX-89898137 in FaFB1 genome

Nine SNP markers, previously located in the 840-kb region of *RXf1* on chromosome 6–2 of the ‘Camarosa’ reference genome, have been relocated to chromosome 6. C of the ‘Florida Brilliance’ (FaFB1) genome [16, 20, 21] (Fig. 1a). Two haplotype 6Ca and 6Cb of FaFB1 were identical for *Rxf1* region. In the FaFB1 genome, two markers (AX-89798089 and AX-89810614) were found to locate outside of the *RXf1* region, whereas the remaining nine markers were relocated in the same order as in 'Camarosa'. In order to track parental contribution for the ALS resistance in progenies, seven new high-resolution melting (HRM) markers, XF_30.893–02, XF_30.901–01, AX-184535293, AX-184491488, XF_30.971–01, XF1HRM02, and AX-184211448, were developed and mapped to the region (Fig. 1b, supplementary Table 1). A total of eight recombinants were identified by genotyping three populations (family 14.100, family 14.101, and the self of FL14.101–225) with six markers (AX-89898263, XF_30.893–02, XF_30.901–01, XF_30.971–01, AX-89798073, and XF1HRM02) (Fig. 1c, supplementary Table 2). Two of these families, 14.100 and 14.101, were generated by Roach et al. and further utilized in this study [16]. In the family 14.100, one recombinant (‘Earliglow’) was identified using the AX-89898263. In the family 14.101, two recombinants (accession FL14.101–220 and 'Earliglow') at the location of AX-89898263, and one (FL14.101-75) at the XF_30.901–01 were identified. By genotyping the FL14.101–225 selfed population, three recombinations (S217, S224, and S225) at the AX-89898263 and two (S1 and S218) at the XF_30.901–01 were identified. According to the recombining marker locations, *RXf1* was redefined as the region spanning from HRM markers, XF_30.901–01 (3.491 Mbp) and AX-89898137 (3.977 Mbp), corresponding to a physical distance of approximately 486-kb interval in FaFB1 (Fig. 1c) containing 54 annotated genes (supplementary Table 3).

### Haplotype-phased genome assembly of ALS resistant accession FL17.68–110

A haplotype-phased genome assembly of FL17.68–110, an ALS-resistant genotype heterozygous at *RXf1*, was assembled. PacBio high-fidelity (HiFi) long-read sequencing produced 2.8 million reads (253 Gb) from a single cell, with an estimated read depth of 8.4 Gb per haploid and N50 of total reads was 185 kb (Supplementary Table 4). By performing hifiasm assembly with the PacBio HiFi long reads, haplotype-specific reads were separated based on the Illumina short reads from the two parents Sweet Sensation® ‘Florida127’ (hereafter referred to as ‘Florida127’) and resistant selection FL14.100–59. A total of 1,031 and 540 contigs were generated for the ‘Florida127’ and FL14.100–59 haplotypes, respectively (Table 1). The size of longest contig was 16.8 Mbp for ‘Florida127’ and 13.7 Mbp for FL14.100–59 (Table 1).

**Table 1:** The summary statistics of FL17.68–110 genome assembly and annotation

	**Haplotype 1** **(‘Florida127’)**	**Haplotype 2** **(FL14.100–59)**
**Assembly Stats**		
Number of Contigs	1,031	540
Total Length	880,544,242	1,022,966,871
Largest Contig	16,848,209	13,711,700
Number of Contigs ≥ 50 kb	404	342
Total length ≥ 50 kb	859,041,470	748,559,761
N50	5,361,504	5,764,495
**Scaffold Stats**		
Number of used Contigs	313	267
Total Length	880,644,442	748,610,861
Largest Contig	70,172,493	34,481,191
Placed bp	810,443,449	729,623,318
Unplaced bp	70,100,793	18,936,443
N50	29,740,329	26,051,279
**BUSCO**		
Complete BUSCOs (C)	99.30%	99.20%
Complete and duplicated BUSCOs (D)	97.20%	97.30%
Fragmented BUSCOs (F)	0.00%	0.10%
Missing BUSCOs (M)	0.70%	0.70%
**Annotation**		
Number of genes	105,795	87,277
Number of proteins	104,984	86,713
Genes with RNAseq support	136,458	112,128
Total size of TE (bp)	418,866,968 (47.57%)	351,369,688 (46.94%)

**Fig. 2:**
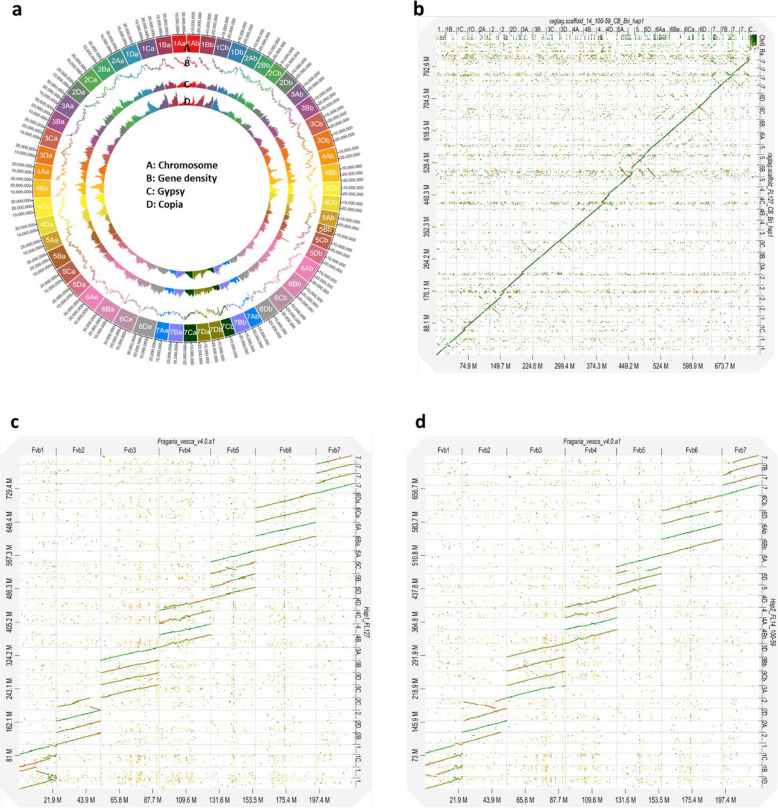
Circos map of important features of genome assembly of FL17.68–110 (**a**) Circos diagram of 56 chromosomes of FL17.68–110 (Genomic variation in two haplotypes of FL17.68–110. The outer track represents chromosomes with unites in megabases. The interior track includes gene density, DNA transposon coverage and retrotransposon coverage. **b** Dot plot depicting the relationships of FL17.68–110 haploid 1 (‘Florida127’) and 2 (FL14.100–59). **c** Collinearity dot plot between *F. vesca* and FL127. **d** Collinearity plot between *F. vesca* and FL14.100–59. Homologous block across haplotypes is connected by lines

Haplotype-separated contigs were scaffolded based on the FaFB1 reference genome using hifiasm (0.16.1-r375). 313 out of 1,031 contigs of ‘Florida127’ and 267 out of 540 contigs of FL14.100–59 were used for building the full haplotypes of 880,644,442 bp and 748,610,861 bp, respectively (Table 1). Compared to the total length from the assembly step, the ‘Florida127’ haplotype showed a similar scaffolded total length, whereas the ‘FL14.100–59’ haplotype exhibited a decreased scaffolded total length (Table 1). The N50 of scaffolded chromosomes were approximately 29,740,329 bp (‘Florida127’) and 26,051,279 bp (FL14.100–59). Overall, the genome of FL17.68–110 consisted of the expected 56 chromosomes, 28 chromosomes from each haplotype (Fig. 2a). The length of each chromosome was similar to FaFB1 and ‘Royal Royce’ (FaRR1) reference genomes [22] (Supplementary Figure S1). The quality and completeness of FL17.68–110 genome was evaluated using BUSCO (v5.2.0) and Merqury (v1.3). The haplotypes scored 99.30% (‘Florida127’) and 99.20% (FL14.100–59) for BUSCO in the eudicot dataset (Table 1). In addition, each scaffolded haplotype was compared to haplotype-specific k-mer (hap-mer) using Merqury (v1.3) (Supplementary Figure S2). The ‘Florida127’ haplotype sequence matched 98.9% with the ‘Florida127’ hapmers and 6.3% matched with FL14.100–59 hapmers. On the other hand, the FL14.100–59 sequence matched 2.4% with the ‘Florida127’ hapmers and 99.2% matched with the FL14.100–59 hapmers. Both haplotypes matched 98.4% with the two hapmers (Supplementary Table 5). The scaffolded genome was masked by ‘RepeatMasker (Version 4.1.1)’ and ‘RepeatModeler (Version 2.0.1)’, resulting in 1,285,293 bp (‘Florida127’) and 1,129,870 bp (FL14.100–59) being masked. The non-masked genome sequences were used for the annotation (Table 1). The scaffolded genome was annotated using GenSAS V6.0 [23]. A total of 105,795 and 87,277 genes were estimated in the ‘Florida127’ and FL14.100–59 haplotpyes (Table 1 and Fig. 2a). In addition, the density of retrotransposons found by Gypsy and Copia had similar patterns over the 56 chromosomes (Fig. 2a). The whole-genome alignment of the two haplotypes showed overall strong collinearity without significant structural variations such as inversions and translocations (Fig. 2b). The two haplotypes were also compared with *Fragaria vesca* whole genome v4.0.a1 (Fig. 2c and d). Comparison with *F. vesca* revealed multiple inversions for chromosomes Fvb1 and Fvb2 (Fig. 2c and d). Notably, analysis of collinearity between *F. vesca *and FL14.100-59 showed higher rates of non-matching regions compared to the ‘Florida127’ haplotype (Fig. 2d). The collinearity with FaFB1 showed high similarity, with the no match area being 4.07% and sequences with over 75% similarity constituting 50.85% in haploid ‘Florida127’ (Supplementary Figure S3a). In haploid FL14.100–59, 10.86% of sequences did not match, and 33.66% of sequences had over 75% similarity (Supplementary Figure S3b).

### Genomic characterization of the *RXf1* region

**Fig. 3:**
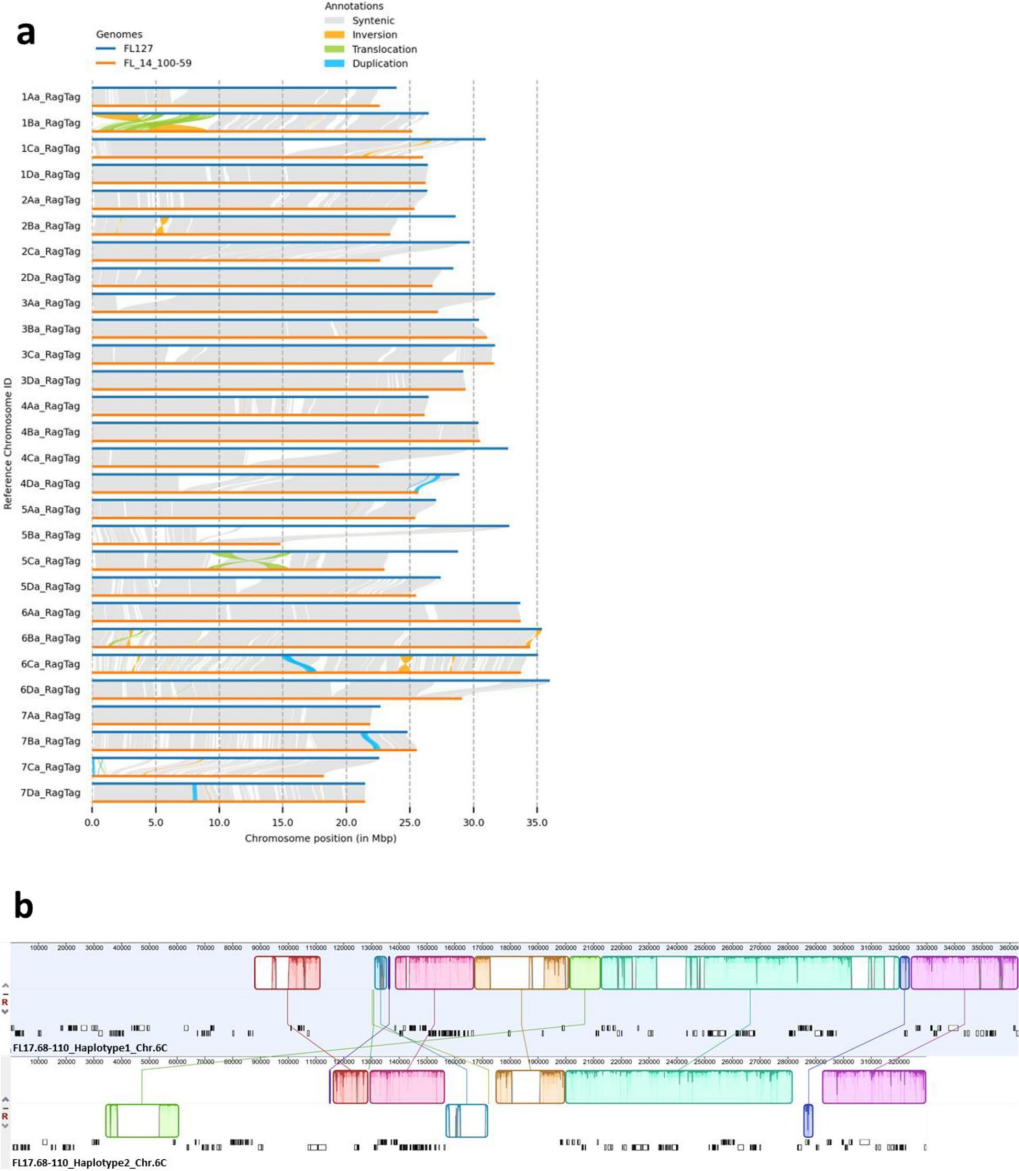
**a** Collinearity map between two haplotypes of the genome assembly, ‘Florida127’ and FL14.100–59. **b** Genomic sequence alignment between ‘Florida127’ and FL14.100–59

Comparative analysis of FL17.68–110 haplotypes ‘Florida127’ and FL14.100–59 showed inversion, translocation, and duplications across the whole genome (Fig. 3a). Chromosome 1Ba showed one-third size of genome region having inversion and translocation, and chromosome 5Ba revealed that the haploid of FL14.100–59 possessed a relatively shorter genome sequence compared to the haploid derived from ‘Florida127’ (Fig. 3a). The 486-kb *RXf1* region also showed nonsyntenic sequences, relocations, and conversion (Fig. 3b). A total of forty-seven genes were in 486-kb region of *RXf1* in FL14.100–59 haplotype genome (resistant – *RXf1*), 52 genes in ‘Florida127’ haplotype genome (susceptible – *rxf1*), and 54 genes were found in ‘FaFB1’ (susceptible – *rxf1*) (Supplementary Table 6). Within the identified genes, fourteen were specific to the FL14.100–59 haplotype genome (Haplotype 2), and fifteen genes were uniquely present in the ‘Florida127’ haplotype genome (Haplotype 1) as outlined in Table 2. Furthermore, approximately 2.5 Mbp of flanking regions upstream and downstream from the *RXf1* region were extracted from eight octoploid strawberry genomes to investigate the broader structural differences across the chromosome-scale reference genomes. All seven varieties (‘Florida Brilliance’ (FaFB1), ‘Royal Royce’ (FaRR1), FL11.46–86, FL12.115–10, Sweet Sensation® ‘Florida127’, Florida Beauty, and FVC11-58), except FL14.100–59, are susceptible to ALS. Nonsystenic region, inversion and translocation of genomic regions were observed between ALS susceptible varieties not only between FL14.100–59 and ‘Florida127’ (Supplementary Figure S4).

**Table 2:** List of genes located in *RXf1* region from two haploids

**Gene**	**Predicted Function**
**Haplotype 1**	
Fa.00g771080.m01	Threonine–tRNA ligase, mitochondrial-like (LOC101306153), transcript variant X3
Fa.00g771090.m01	Protein ENHANCED DISEASE RESISTANCE 2 (LOC101306446), transcript variant X2
Fa.00g771100.m01	Rosa chinensis protein FAR1-RELATED SEQUENCE 5 (LOC112194297), transcript variant X3
Fa.00g771120.m01	SNF1-related protein kinase catalytic subunit alpha KIN10 (LOC101307030)
Fa.00g771130.m01	SNF1-related protein kinase catalytic subunit alpha KIN10 (LOC101307030)
Fa.00g771140.m01	Endo-1,3(4)-beta-glucanase 2-like (LOC101314783)
Fa.00g771150.m01	Rosa chinensis putative endo-1,3(4)-beta-glucanase 2 (LOC112186895)
Fa.00g771160.m01	Fragaria x ananassa transcription factor ERF42
Fa.00g771170.m01	CSC1-like protein HYP1 (LOC101307320)
Fa.00g771180.m01	Vesicle transport v-SNARE 13 (LOC101307608)
Fa.00g771220.m01	Probable disease resistance protein At4g27220 (LOC101303577), transcript variant X3
Fa.00g771230.m01	Pentatricopeptide repeat-containing protein At5g15280-like (LOC101293811)
Fa.00g771260.m01	B3 domain-containing transcription factor LEC2-like (LOC101298714), transcript variant X2
Fa.00g771300.m01	Putative F-box protein At4g22170 (LOC101304316), transcript variant X1
Fa.00g771380.m01	Uncharacterized LOC101301069 (LOC101301069), transcript variant X5
**Haplotype 2**	
Fa.00g675590.m01	Putative proline–tRNA ligase C19C7.06 (LOC101307525)
Fa.00g675600.m01	Membrane-anchored ubiquitin-fold protein 1 (LOC101300166), transcript variant X4
Fa.00g675610.m01	Probable galacturonosyltransferase 14 (LOC101299885), transcript variant X2
Fa.00g675620.m01	Nucleosome assembly protein 1;3-like (LOC101307238), transcript variant X5
Fa.00g675630.m01	Nucleosome assembly protein 1;3-like (LOC101307238), transcript variant X5
Fa.00g675640.m01	Uncharacterized LOC105352885 (LOC105352885), transcript variant X3
Fa.00g675670.m01	Rosa chinensis tyrosine–tRNA ligase 1, cytoplasmic (LOC112184062)
Fa.00g675680.m01	25.3 kDa vesicle transport protein-like (LOC101298530)
Fa.00g675690.m01	DNA-directed RNA polymerase I subunit rpa49-like (LOC101306367)
Fa.00g675700.m01	BAG family molecular chaperone regulator 8, chloroplastic (LOC101298238)
Fa.00g675730.m01	Uncharacterized LOC101305788 (LOC101305788)
Fa.00g675740.m01	U5 small nuclear ribonucleoprotein 200 kDa helicase-like (LOC101305204)
Fa.00g675990.m01	Uncharacterized LOC101297658 (LOC101297658), transcript variant X3
Fa.00g676000.m01	40S ribosomal protein S9-2 (LOC101295733)

### BAC library screening for identifying clones associated with *RXf1*

To obtain the genomic sequence for ALS resistance, resistance genotype FL14.101–225 containing *RXf1* were used for BAC screening and cloning process. Through the screening of three BAC libraries with eleven markers, a total of 625 BAC clones were identified, out of which 235 clones were detected in the ALS-resistant genotype FL14.101–225 library, and 182 and 208 clones were detected in the ALS-susceptible FL11.77–96 and ‘Florida Brilliance’ libraries, respectively (Supplementary Table 7 and 8). Finally, eight BAC clones were selected to sequence using four markers (AX-89898194, AX-89798073, AX-89898263, and C107-2) that are specific to subchromosome 6–2 in the ‘Camarosa’ genome, which corresponds to chromosome 6C in the FaFB1 and FaRR1 genomes. Pair-end Illumina high-throughput sequencing produced between 1.8 to 2.63 clean base (G) data for all eight clones, respectively (Genewiz, South Plainfield, NJ, USA). The physical location of each BAC clone was identified in the FL14.100–59 genome, and 276,838 bp out of a 636,884 bp region were mapped with the BAC contigs onto the FL14.100–59 genome (Supplementary Figure S5).

### Transcriptome analysis to identify candidate genes associated with *RXf1*

**Fig. 4:**
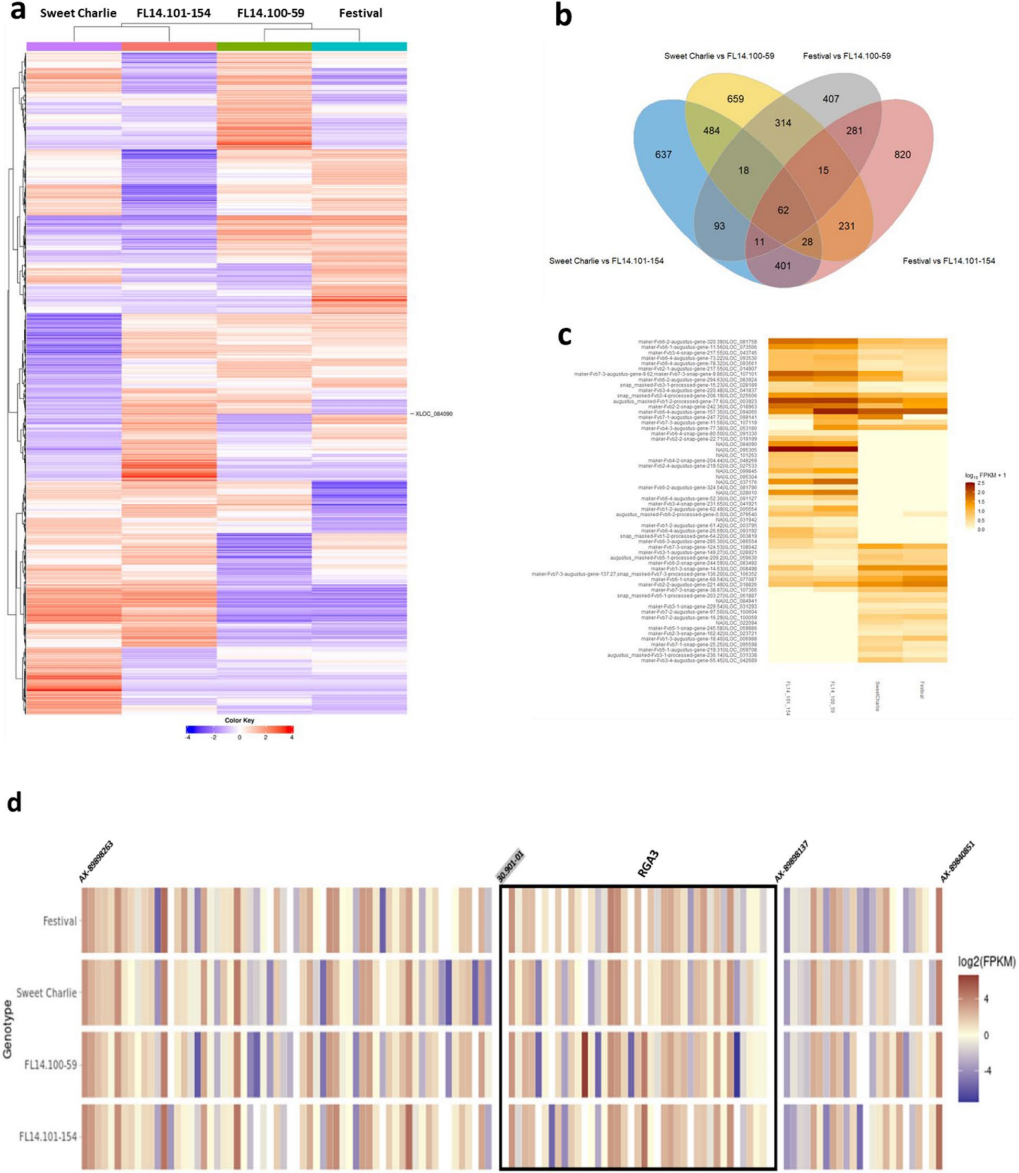
RNA-seq analysis between ALS-resistant and susceptible genotypes **a** Whole genome-wide heatmap in four genotypes. **b** Venn diagram indicates four comparisons between resistant and susceptible genotypes. **c** Heatmap of 62 DEGs from four comparisons from Venn diagram **d** Heatmap of the genes located within 486-kb *RXf1* region in four genotypes

RNA-Seq analysis was performed by analyzing data at two time points (48 and 96-hpi) separately in each cultivar and breeding selection: ‘Strawberry Festival’, ‘Sweet Charlie’, FL14.100–59, and FL14.101–154. The gene expression patterns in the four genotypes were shown in the whole genome-wide heatmap (Fig. 4a). In the hierarchical clustering analysis among varieties, the DEG patterns for ‘Sweet Charlie’ and FL14.101–154 showed proximity, while ‘Strawberry Festival’ and FL14.100–59 are clustered together. From each genotype about 65,000 out of 112,199 genes in the whole genome were expressed, and about 56,000 genes were commonly expressed in all four varieties. To identify the DEGs between the four varieties, pairwise comparisons were conducted. A total of 10,285 significant DEGs were identified from six comparisons containing 5,176 upregulated and 5,109 downregulated genes (Supplementary Figure S6a). Among all DEGs, a total of 62 genes comprising 51 annotated genes and 11 genes without annotation were found to overlap in all four comparisons (Fig. 4b). The expression of 62 genes was shown in Fig. 4c, in which 21 genes were highly expressed in two resistant breeding selections compared to the two susceptible cultivars. The top twenty pathways with the DEGs included lyase activity, development related pathways, phosphoprotein, transmembrane transport, and nucleotide binding relate pathways (Supplementary Figure S6b). The expression of genes located at a 486-kb region of *RXf1* in four accessions were shown in Fig. 4d.

A total of 177 genes were located within the 607-kb *RXf1* region which is the size of *RXf1* before fine-mapping. 40 genes located at 486-kb region of fine-mapped *RXf1* region. Only one gene was found to be significant DEG in ALS-resistant selections compared to the susceptible varieties, and this gene was functionally annotated as *resistance gene analogue 3* (*RGA3*) in *F. vesca*. The *RGA3* was categorized into a leucine-rich repeat pathway, however, this pathway was not within the top 20 pathways in terms of enrichment level. The gene expression level of *FaRGA3* was confirmed by reverse transcript quantitative PCR (RT-qPCR) with the housekeeping gene *FaPDH* gene. The expression of *FaRGA3 *increase 48 h after inoculation in resistant genotypes, FL14.100–59 and FL14.101–225 (Supplementary Figure S7).

The genomic structure of *FaRGA3* from ALS-resistant allele showed the 3,820-bp insertion in the intron region between the second exon and the third exon, while the susceptible allele did not have the insertion between exons (Supplementary Figure S8).

## Discussion

Breeding strawberry varieties with desirable traits including flavor, yield, plant architecture and disease resistance poses a number of challenges. In order to combine many desirable traits with disease resistance, tools and knowledge are needed to make the breeding process more efficient and precise. In this study, we present the fine-mapping of *RXf1* and a haplotype-phased genome assembly of the elite ALS-resistant accession. Combined with transcriptomic analysis, these resources allowed for the identification of a candidate resistance gene that could be the target of precision breeding approaches.

In previous studies, two ALS resistance sources, US4808 and US4809, were identified in octoploid strawberry, while multiple sources were detected in non-octoploid strawberry species, *F. pentaphylla* and *F. moschata* [14, 24]. The two resistant octoploid sources were introgressed into Agriculture and Agri-Food Canada (AAC) and then University of Florida strawberry germplasm, and four segregating populations for resistance were generated [13, 14, 16]. In previous study, the *RXf1* region was defined to approximately 500 kb (family 13.77) and 900 kb (family 13.78) regions of linkage group 6D in *Fragaria vesca* spp. vesca and *F. vesca* spp. bracteate reference genomes [16, 25, 26]. Subsequently, the *RXf1* locus was confined to the 840 kb region in the ‘Camarosa’ reference genome (30.52 – 31.36 Mbp at chromosome 6–2). Additionally, the physical location of *RXf1* was defined by nine IStraw90 Axiom® array SNP markers (AX-89898263, AX-89798107, AX-89840981, AX-89798089, AX-89810614, AX-89798073, AX-89898194, AX-89898137, and AX-89840851) in the ‘Camarosa’ genome [16–18, 20]. However, the precise location of *RXf1* and the number of genes involved could not be determined due to the size of the region.

The previous SNP marker information was based on the *F. vesca* and *F.* × *ananassa* ‘Camarosa’ reference genomes. We relocated the SNP markers to recent high-quality reference genome FaFB1 and developed HRM markers [19]. A new marker for breeding, XF1HRM2, which was designed based on one SNP and a 6-bp insertion, replaced previous marker XF1HRM1 (AX-89898194) used for ALS resistance selection via marker-assisted seedling selection (MASS). The two markers are separated by 142,200 bp, and candidate gene *FaRGA3* is 85,270 bp from XF1HRM2.

Our work focused on constructing genomic sequence for *RXf1* and identifying candidate genes for resistance to angular leaf spot in octoploid strawberry. In this study, we completed the genomic sequence of *RXf1* using two approaches: haplotype-phased whole genome assembly and BAC sequencing. The result showed identical sequences between whole genome assembly and BAC sequencing. To scaffold contigs from two haplotypes of FL17.68–110, the FaFB1 reference genome was used. As resistance to *X. fragariae* was derived from *F. virginiana*, reference-guide scaffolding was also conducted using the assembled *F. virginiana* genome [27]. Overall, most chromosomes from both genomes, *F.* × *ananassa* and *F. virginiana*, generated haplotypes of similar length. However, when *F. virginiana* was used as the reference genome, several chromosomes from both haplotypes resulted in longer sequences, while a few *F.* × *ananassa*-guided chromosomes produced the longest sequences (Supplementary Figure S9). Despite changing the reference genome for scaffolding, comparatively shorter chromosome lengths were still observed in chromosome 2B, 4C, 5B, and 7C from haploid FL14.100–59 (Supplementary Figure S9). Based on these results, it was found that the missing pieces from shorter chromosome were still present. To verify whether these regions were omitted due to the use of FaFB1, the unscaffolded contigs from scaffolding with FaFB1 were aligned with the *F. virginiana* genome. Alignment results showed that the small unscaffolded contigs aligned broadly across the chromosomes of *F. virginiana*, with approximately 7 Mbp of unscaffolded contigs from 'Florida127' mapping to chromosome 5B and 2 Mbp of the contigs from FL14-100–59 mapping to chromosome 5A (Supplementary Figure S3c and d). This result may suggests that chromosome 5B from the FL127 haplotype and chromosome 5A from FL14-100–59 contain introgressed *F. virginiana* genome segments. A comparison of the *RXf1* region between the two reference genomes used for scaffolding revealed that no differences in genomic sequences or structural variations between the two scaffolding results (Supplementary Figure S10). Therefore, increasing the number of cells for sequencing, sequencing depth, and incorporating long-read sequencing (Hi-C) data may further enhance the completeness of assembly of FL17.68–110.

The most promising candidate gene is a putative disease resistance protein *Resistance gene analogue 3* (*RGA3*). Resistance gene analogue (RGA) is a predicted disease resistance gene (*R* gene) that encodes nucleotide-binding site leucine-rich repeat (NBS-LRR) combined with different motifs such as Toll/interleukin-1 receptor-like domain or coiled-coil domain [28]. In diverse plant species including strawberry, RGAs were searched and predicted by conserved sequences in the NBS domain [29–31]. According to gene annotation, *RGA3* encodes coiled-coil domain followed by NBS-LRR domains, which are involved in pathogen recognition. Those findings suggest that *FaRGA3* is a typical *R* gene that may confer ALS resistance in strawberry. Additionally, the expression of immunity-related marker genes, *PR1* (*Pathogenesis-Related 1*) and *FRK1* (*Flagellin Receptor Kinase 1*), was confirmed through RNA-seq analysis. An orthologous gene search was conducted in *Fragaria vesca* using Arabidopsis genes. Based on the best BLAST match scores, three orthologous genes for *PR1* (a) and *FRK1* (b) were identified. However, no unique patterns were observed that were specific to either resistant or susceptible genotypes. (Supplementary Figure S11).

A hypersensitive reaction (HR) was confirmed by inoculating two concentrations of *X. fragariae* into ALS-resistant plants, while no HR symptoms were observed in ALS-susceptible cultivars (Supplementary Figure S12a). Furthermore, transient overexpression of *FaRGA3,* combined with inoculation of *X. fragariae* and *X. perforans* was performed in *Nicotiana benthamiana*, resulting in increased reactive oxygen species (ROS) as confirmed by diaminobenzidine tetrahydrochloride (DAB) staining (Supplementary Figure S12b). Based on these results, further studies involving stable gene expression are highly recommended for validating gene function.

Potential allelic diversity at the *RXf1* locus is a relevant subject because family 14.100 and 14.101 were generated from two different resistance resources. The genotype US4808 is collected from a native *F. virginiana* from Minnesota, and US4809 is a hybrid between *F. virginiana* from Georgia and *F.* × *ananassa* `Earliglow' [14]. Although both genotypes are *F. virginiana*, they are different accessions. In the transcriptome analysis of FL14.100–59 and FL14.101–154 after the pathogen infection, we observed no distinct gene expression patterns among the two sources. Furthermore, we conducted two methods to construct the genomic sequences of *RXf1* in genotypes from family 14.100 and 14.101. The sequences of BAC contigs from FL14.101–225 were perfectly aligned with the FL14.100–59 sequence except for few SNPs apparently caused by sequencing errors. The genomic sequences *FaRGA3* also perfectly matched between the two genotypes (Supplementary Figure S13). This result strongly suggests that there is no allelic difference for *RXf1* and *FaRGA3* between the two resistant sources, suggesting a single resistant allele in octoploid strawberry.

## Conclusions

Our findings strongly suggest that *FaRGA3* (*Resistance Gene Analogue 3*) plays a significant role in controlling disease resistance against *Xanthomonas fragariae* in octoploid strawberry. Additionally, our results confirm the identical genomic sequences of two integrated ALS resistance sources for *FaRGA3* and the fine-mapped region. The highly specific indel markers developed in this study can contribute to the selection of ALS-resistant parents and seedlings in strawberry breeding programs. Furthermore, the phased genome assembly of the ALS-resistant accession FL17.68–110 could be a valuable resource for genomic research in cultivated strawberries. In future research, functional validation and characterization of *FaRGA3* will be important. Thus, we will aim to explore functional mechanisms of resistance and develop additional breeding and genetics resources for ALS resistance in strawberry.

## Methods

### Plant materials

Three populations comprised of 663 individuals were utilized for fine mapping of *RXf1*. Two full-sib families 14.100 (*n* = 125) and 14.101 (*n* = 287) were prepared, which were derived from crosses between ALS-susceptible and -resistant accessions (FL11.28–34 (S) × FL13.78–57 (R) and ‘Florida 127’ (S) × FL13.77–5 (R)) in the previous study (Supplementary Figure S14) [16]. Phenotypic data and genomic information obtained by Roach et al. (2016) were reanalyzed for this study [16]. Additionally, a first selfed generation (*n* = 242) was generated from ALS-resistant accession FL14.101–225 (Supplementary Figure S14). FL14.101–225 plants were grown and self-pollinated in the greenhouse at the UF Gulf Coast Research and Education Center (GCREC) in Wimauma, Florida. Seeds were harvested from fully ripe fruits and germinated on Murashige and Skoog media (MS) medium containing 0.5% (w/v) of TC agar after seed scarification with sulfuric acid. Two-week old seedlings were transplanted into peat pallets and grown for one month in the greenhouse.

To conduct RNA-seq, four genotypes including two susceptible (‘Strawberry Festival’ and ‘Sweet Charlie’) and two resistant (FL14.100–59 and FL14.101–154) individuals were prepared in the greenhouse and grown for a month after runner propagation.

To build a high-quality phased genome having ALS resistance, heterozygous ALS-resistant accession FL17.68–110 was selected based on its durable resistance to bacterial angular leaf spot. This selection was derived from a cross between FL14.100–59 (ALS-resistant) and Sweet Sensation® 'Florida 127' (ALS-susceptible). Octoploid strawberry accession FL17.68–110 (*F.* × *ananassa*) was grown in the breeding field trials of the Gulf Coast Research and Education Center in Wimauma, Florida. The plants were etiolated for 10 days, and young etiolated leaf tissues were collected and 1 g of leaf was used for high molecular weight DNA isolation.

### *X. fragariae* inoculation

Phenotyping data for two full-sib families 14.100 (*n* = 125) and 14.101 (*n* = 287) was obtained from Roach et al. [16]. To phenotype individuals from the selfed population, four *X. fragariae* isolates collected from naturally infected strawberry fields and nursery were used for inoculation. Two isolates, xf06-80 and xf06-81, were collected in 2006 from a commercial farm, Ferris Farms, in Citrus County, Florida, from the host cultivar ‘Festival.’ The other two isolates, xf09-20 and xf09-32, were collected in 2009 from the Gulf Coast Research and Education Center (GCREC) in Hillsborough County, Florida, from the host cultivars ‘Festival’ and ‘Ventura.’ These isolates were confirmed using XF9-XF11 PCR markers (Supplementary Table 9). The inoculum was grown on Wilbrink’s medium (WB) for four days at 28 °C and diluted to OD600 = 0.2 with sterile water [32]. The inoculum was prepared by equally mixing the four isolates in identical ratio. A working inoculum containing 0.005% of Silwet® L-77 was sprayed on plants that were at 3 – 5 leaf stage until each side of the leaf was fully wet. The inoculated plants were covered with transparent vinyl bags for 10 days post-inoculation (dpi) to maintain a fully saturated conditions, and phenotyping was conducted five times from 10-dpi at 2-day intervals in the second and third youngest leaves from the top of each plant. Individual plants were scored for percent diseased leaf area on five scales from 0 to 100% with 20% increment, and leaf areas with less than 40% disease coverage were scored as resistant, while those with over 40% coverage were scored as susceptible to *X. fragariae* (Supplementary Figure S15).

To prepare samples for RNA-seq, four genotypes were inoculated as previously mentioned. Control plants were treated with sterile water instead of inoculum. Both inoculated and control plants were also covered with transparent vinyl bags for fully saturated conditions. Leaf tissues were collected at 0, 12, 24, 48, 72, and 96 h post-inoculation (hpi) during pathogen infection and stored at −80 °C until use. The phenotyping was conducted from 10 to 14 dpi.

### DNA and RNA isolation

Unexpanded trifoliate leaves were collected from the inoculated plants and the samples were stored at—80 °C. The frozen samples were ground finely using a PowerGen™ homogenizer (Thermo Fisher Scientific, MA, USA) with three 4 mm diameter glass beads. Genomic DNA was extracted from 100 mg of ground tissues using a modified CTAB method [33, 34]. The DNA concentration was confirmed using a NanoDrop™ 8000 Spectrophotometer (Thermo Fisher Scientific, MA, USA), and the original DNA was diluted to 50 ng/ul with 1X TE solution (pH 7.5, Integrated DNA Technologies, IA, USA) for genotyping.

For the RNA-seq samples, the collected leaf samples were finely ground using mortar and pestle with keeping a frozen condition. Total RNA of ground samples was extracted using Spectrum™ Plant Total RNA Kit (Sigma-Aldrich, MO, USA) followed by the manufacturer’s instructions. Three replicates were pooled into one sample with equal amounts. RNA concentration was measured using a NanoDrop™ 8000 spectrophotometer (Thermo Fisher Scientific, MA, USA) and extracted RNA was diluted with RNase-free water.

### High resolution melting curve (HRM) molecular marker

SNP-based HRM markers were developed based on IStraw90 Axiom® array and *F.* × *ananassa*-based 50 K SNP array (‘FanaSNP’) information [17, 19]. Among newly designed seven HRM markers, four HRM markers, XF_30.893–02, XF_30.901–01, XF_30.971–01, and XF1HRM02, were designed based on BAC contig sequences of ALS-resistant genotype FL14.101–225, and three markers, AX-184535293, AX-184491488, and AX-184211448, were added after the new ‘FanaSNP’ was available [19] (Fig. 1b). PacBio sequencing contigs of ALS-resistant genotype FL14.100–59 were also utilized for developing markers. The location and specificity of the designed primers were confirmed in the octoploid strawberry ‘Camarosa’ and ‘Reikou’ genomes [19, 35, 36].

High-throughput genotyping was performed in the three prepared populations using LightCycler ® 480 system (Roche Life Science, Penzberg, Germany) with the following PCR condition: 94 °C for 3 min, followed by 35 cycles of 94 °C for 30 s, 62 °C for 30 s and 72 °C for 30 s. The PCR reaction was conducted with 2X AccuStart II PCR SuperMix (Quantabio, MA, USA) followed by the manufacturer’s protocol.

### BAC library screening

The BAC library construction was carried out by Amplicon Express (Pullman, WA, USA). Three genotypes, FL 11.77–96, ‘Florida Brilliance’, and FL14.101–225, were used to construct BAC libraries. The DNA from each genotype was digested with two restriction enzymes, BamH1 and HindIII. The resulting 115 – 180 kb of recombinant DNA fragments were inserted into vector pCC1 BAC (EPICENTRE, Madison, WI, USA) and transformed to the *Escherichia coli* (*E. coli*) Phage Restraint DH10B per genotype. Each BAC library comprised 18 superpools, each of which was made up of 23 matrixpools. Nine IStraw90 Axiom® array SNP markers were used to screen BAC clones in the *RXf1* region. The superpool screening was conducted with the following PCR condition: 94 °C for 3 min, followed by 35 cycles of 94 °C for 30 s, 61 °C for 30 s and 72 °C for 30 s. PCR results were checked by running electrophoresis in the 1% agarose gels stained with SYBR® safe DNA gel stain (Invitrogen, MA, USA). Matrixpools detected from superpool screening were screened in the LightCycler ® 480 system (Roche Life Science, Penzberg, Germany) with the identical PCR condition described previously. The PCR reactions were conducted with 2X AccuStart II PCR SuperMix (Quantabio, Beverly, MA, USA) according to the manufacturer’s protocol. The identified BAC clones were grown in Luria broth (LB) medium containing 20 μg/mL of chloramphenicol, and plasmid DNA was extracted using Zyppy Plasmid Miniprep kit (Zymo Research, Irvine, CA, USA) for sequencing confirmation.

A total of eleven primer sets were used for BAC clone screening, and the positive PCR control primer set, AM001-C12-M13, was used according to the provider’s recommendation (Supplementary Table 7). Eight BAC clones were from ALS-resistant accession FL14.101–225 library. The Sanger BAC-End sequencing was conducted using T7 and M13 primers (GENEWIZ, South Plainfield, NJ, USA). The Sanger sequencing data was blasted to the chromosome 6 and confirmed BAC clones were subgenome specific to 6–2 in ‘Camarosa’ genome. Next-generation sequencing of the eight clones was employed to generated BAC contigs of size ranging from 40 – 80 kb, which were aligned to the ‘Camarosa’ reference genomes.

The plasmid DNA of the identified BAC clones was extracted using QIAGEN® Plasmid Mini Kit (QIAGEN, Valencia, CA, USA). The concentration and quality of extracted plasmid DNA were assessed using Nanodrop 8000 Spectrophotometer (Thermo Fisher Scientific, MA, USA). Sanger sequencing was conducted to confirm the beginning sequence of insert using T7 and M13 primers (GENEWIZ, South Plainfield, NJ, USA). After confirmation, BAC plasmid DNA was re-extracted using the QIAGEN® Plasmid Midi Kit (QIAGEN, Valencia, CA), and next-generation sequencing (NGS) was conducted by Illumina hPE150 sequencing (NOVOGENE, Chula Vista, CA, USA). Adapter sequences and low-quality reads were trimmed from the raw sequenced reads of BAC clones, and de novo assembly was performed with the trimmed data using Geneious Prime 2019.2.1 (https://www.geneious.com) and CLC Genomics Workbench 10 (https://digitalinsights.qiagen.com). The de novo assembled contigs were aligned to ‘Camarosa’ genome to confirm the location of contigs in *RXf1* region.

### Next generation sequencing for whole genome assembly

High molecular weight DNA of FL17.68–110 was extracted for long-read sequencing by DNA Link, Inc (Seoul, South Korea). The HiFi reads of FL17.68–110 were generated with one HiFi SMRT cell sequencing using Sequel II platform (Pacific Biosciences Inc., CA, USA) and the data contained 37 gigabyte (Gb) of sequence in 2.8 M reads.

We used a total of four 150-bp paired-end (PE150) Illumina WGS short-read sequences from FL17.68–110 maternal parent Sweet Sensation® ‘Florida127’ and paternal parent FL14.100–59 to perform trio-binning of FL17.68–110’ HiFi sequences. From ‘Florida127’ about from 22 to 27 Gb data was produced in 71,944,741 and 80,186,196 reads. FL14.100–59 produced 65,364,702 and 71,062,368 reads. All the raw sequence data were assessed with ‘FastQC’ (https://www.bioinformatics.babraham.ac.uk/projects/fastqc/) and ‘LongQC’ [37] to confirm the quality of the raw sequences. Adapter trimming steps were conducted before assembly.

### Genome assembly and phasing

Genome assembly, phasing, and annotation were conducted using the University of Florida Research Computing system (http://researchcomputing.ufl.edu). The high-quality and long-read sequence data generated using PacBio Sequel II platform were adapter-trimmed and assembled using contig-scaled trio-binning from ‘hifiasm’ version 0.12-r304 with default parameters [38]. To construct haplotype-phased sequences for each haplotype of FL17.68–110, PacBio long-read sequences of ‘Florida127’ and FL14.100–59 were used to separate contigs. The ‘yak’ version 0.1-r58-dirty was used to identify haplotype-specific 19-nt kmers in the parental short-read sequences (https://github.com/lh3/yak). After trio-binning based on the parental Illumina sequences, the contigs were assessed for quality using ‘Quast’ version 5.0.2 to confirm the assembly results [39]. The quality of trio-binned assembled contigs was assessed with ‘BUSCO’ v5.2.0 in eukaryote pool [40]. With the assembled contigs, reference-guided scaffolding was conducted based on the high-quality of ‘Florida Brilliance’ reference genome using ‘hifiasm’ v2.0.1 [21]. To assess the phased chromosome-assembled genome, ‘merqury’ version 1.1 was used to conduct k-mer pooling for the parental sequences and progeny sequence, and the sequence specificity was evaluated with the scaffolded genome sequences.

### Annotation and gene prediction

The annotation of the FL17.68–110 genome was conducted using the online platform Genome Sequence Annotation Server (GenSAS) [23]. A repeat library was generated from the chromosomal scaffolded genome using ‘RepeatMasker’ and ‘RepeatModeler’ [41, 42]. RNA-seq data was mapped to the assembled genome to annotate protein-coding genes. Additionally, DIAMOND was used to annotate the assembled genome against protein reference databases [43]. Gene prediction was conducted using Augustus, BREAKER, and GeneMarkES. Multiple prediction information was combined using EvidenceModeler, and the BUSCO score of the consensus gene set was assessed. For gene function annotation, blastp, InterProScanm, Pfam, SignalP, and TargetP were used. For comparative genomic sequence analysis, alignment of four genomes (‘FaFB1, ‘Florida127’, FL14.100–59, and ‘FaRR1’) was performed using Mauve aligner [44] (Supplementary Figure S16). The alignment results consisted of locally collinear blocks that indicate the homologous region shared by sequences and no rearrangements between sequences. Each XF_30.901–01 and AX-89898137 marker are located at the left and right border of the alignment result.

### cDNA library construction and RNA-seq analysis

A total of 16 samples collected from four genotypes, two different time points (48 and 96-hpi), and two conditions (inoculation and water control) were prepared for the cDNA library construction (Novogene Corporation Inc, CA, USA). The sequencing of each sample was performed using the Illumina system to produce 75 paired-end sequences (Illumina PE75, 20 M reads per sample).

The RNA sequencing analysis was conducted using the most recent version of the *Fragaria* × *ananassa* 'Camarosa' Genome v1.0 available at the time. RNA sequencing data were analyzed using Tuxedo software suite [45, 46]. Pair-end sequences were aligned using Bowtie2 (2.3.4.3), and aligned sequences were mapped to the octoploid strawberry ‘Camarosa’ genome using TopHat (v2.1.2). Differential gene expression analysis was performed with Cufflilnks and CummeRbund packages. The read counts were normalized with the value of fragments per kilobase of transcript per million mapped reads (FPKM). Based on the normalized value, differential expressed genes (DEGs) analysis was conducted, and the genes were considered as significant when the p-value is less than 0.05 and the fold change is over 2. Venn diagrams were drawn to visualize the differentially expressed genes (DEGs) data using InteractiVenn [45, 47]. All the analyses were performed on HiPerGator Research Computing interspaces (http://researchcomputing.ufl.edu) and R studio ‘1.3.959’. The DEGs were blasted in the *Arabidopsis thaliana* database for functional annotation using ‘Tripal MegaSearch’ in the Genome Database for Rosaceae (GDR). Gene ontology analysis was conducted using ShinyGO 0.77 (http://bioinformatics.sdstate.edu/go/).

To identify candidate genes for ALS resistance, transcriptome analysis was performed based on RAN-seq results across the whole genome of octoploid strawberry ‘Camarosa’. RNA-seq generated an average of 28 million raw reads per sample, and 64.6–85.9% of the raw reads from samples were mapped to the ‘Camarosa’ genome. The average number of aligned pairs was 19 million, and the rate of concordant pair alignment was 63.6% on average (Supplementary Table 10).

## Supplementary Information

Supplementary Material 1.

Supplementary Material 2.

## Data Availability

Raw transcriptome data of this study will be available in National Center for Biotechnology Information (NCBI) Sequence Read Archive (SRA) (https://www.ncbi.nlm.nih.gov/sra) using study ID (PRJNA1135936). Assembled genome of FL17.68–110 can be found on Genome Database for Rosacea (GDR) (https://www.rosaceae.org/) with accession number (tfGDR1080).
