# Supplementary material for: A haplotype-phased genome characterizes the genomic architecture and causal variants for RXf1 conferring resistance to Xanthomonas fragariae in strawberry (F. × ananassa)
Source: BMC Genomics. 2025 May 8;26:453. doi: 10.1186/s12864-025-11517-w (PMC12060388; doi:10.1186/s12864-025-11517-w)
Supplement: Supplementary file 2 — Supplementary Material 2. [file 12864_2025_11517_MOESM2_ESM.pdf]

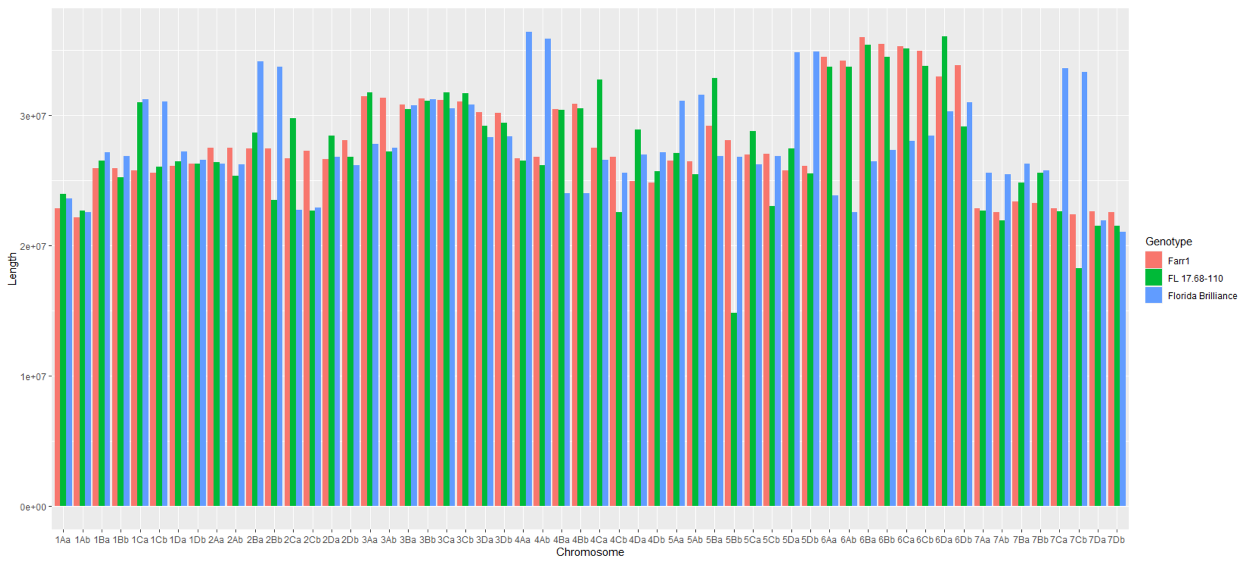

Supplementary Figure S1. Length of chromosome among three genomes; Royal Royce (Farr1), FL17.68-110 and 'Florida Brilliance' (FaFB1).

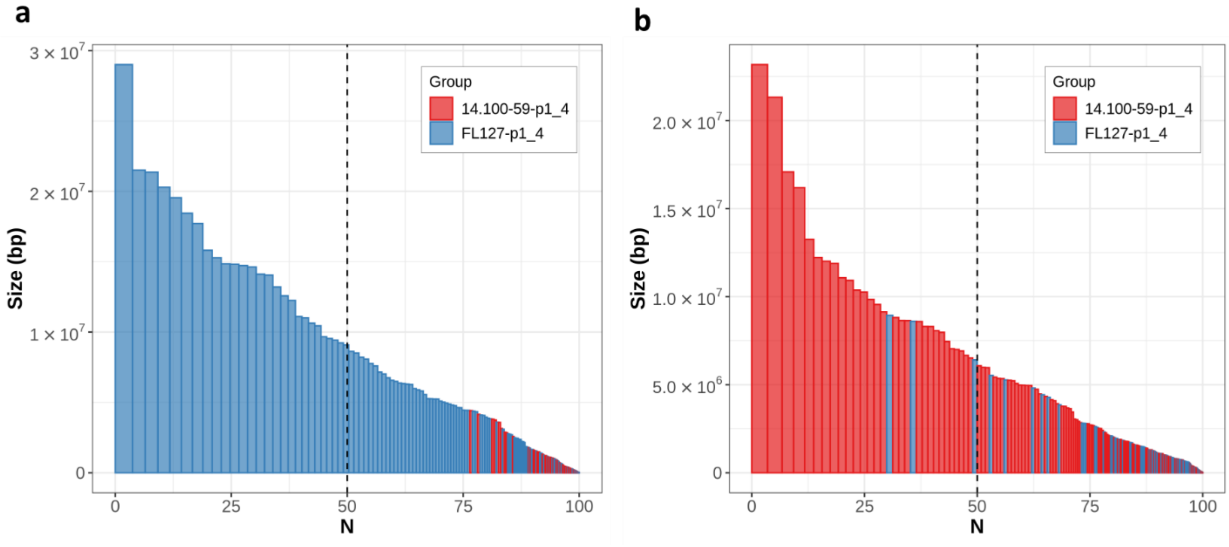

Supplementary Figure S2. Phase block plots for two haploids of FL17.68-110, ‘Florida127’ (FL127, **a**) and FL14.100-59 **(b)**, using Merqury. Most haplotype blocks are matched with the k-mer group from the corresponding haplotype, and blocks from incorrect haplotypes are found as small entities.

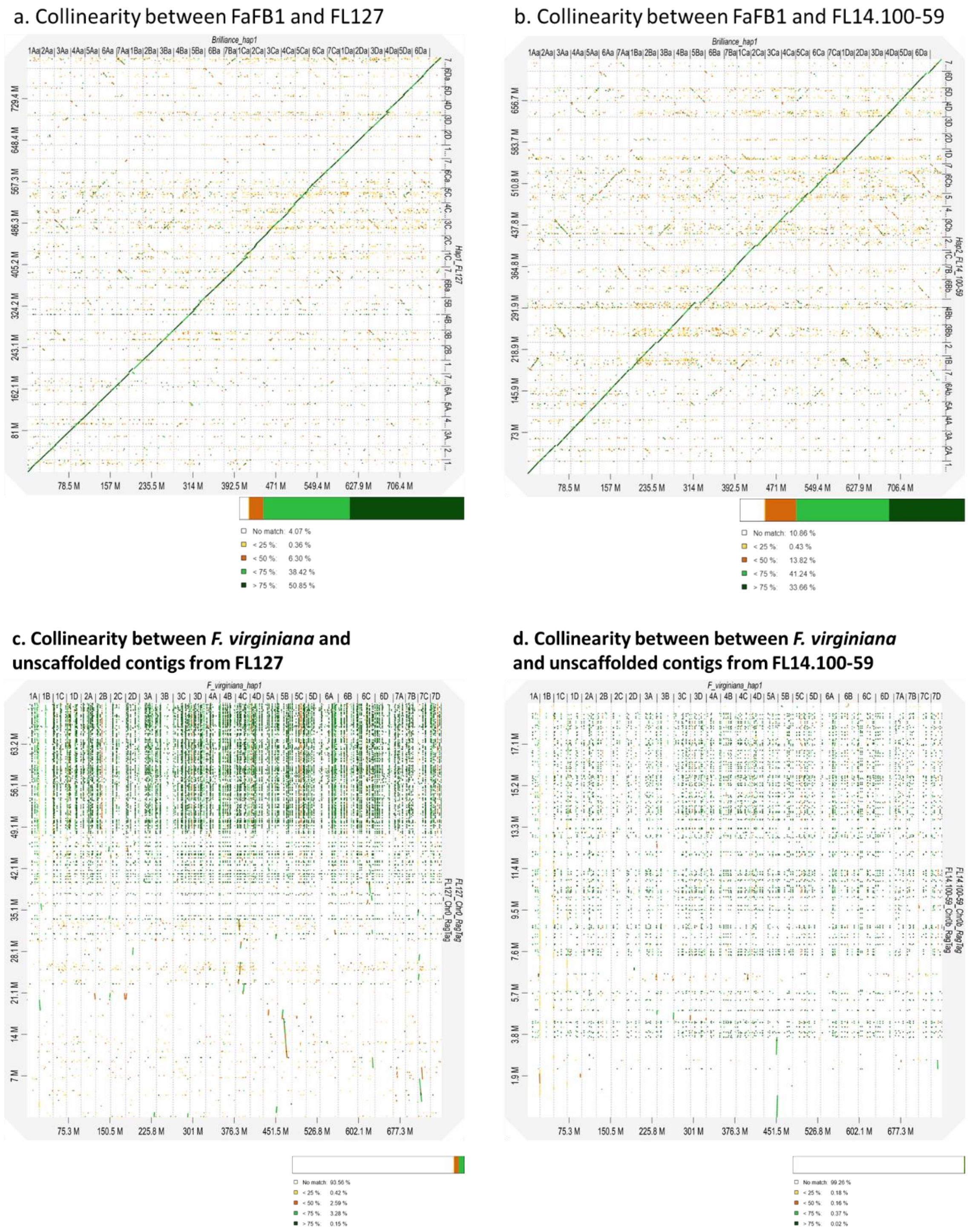

Supplementary Figure S3. Dot plots generated comparing two haploids of FL17.68-110 to *Fragaria ananassa* and *F. virginiana*. Brilliance (FaFB1) was compared with Haploid 1 **(FL127, a)** and Haploid 2 **(FL14.100-59, b)**. Additionally, dot plots illustrate the comparison of *F. virginiana* with unscaffolded contigs from FL127 (**c**) and FL14.100-59 (**d**), respectively.

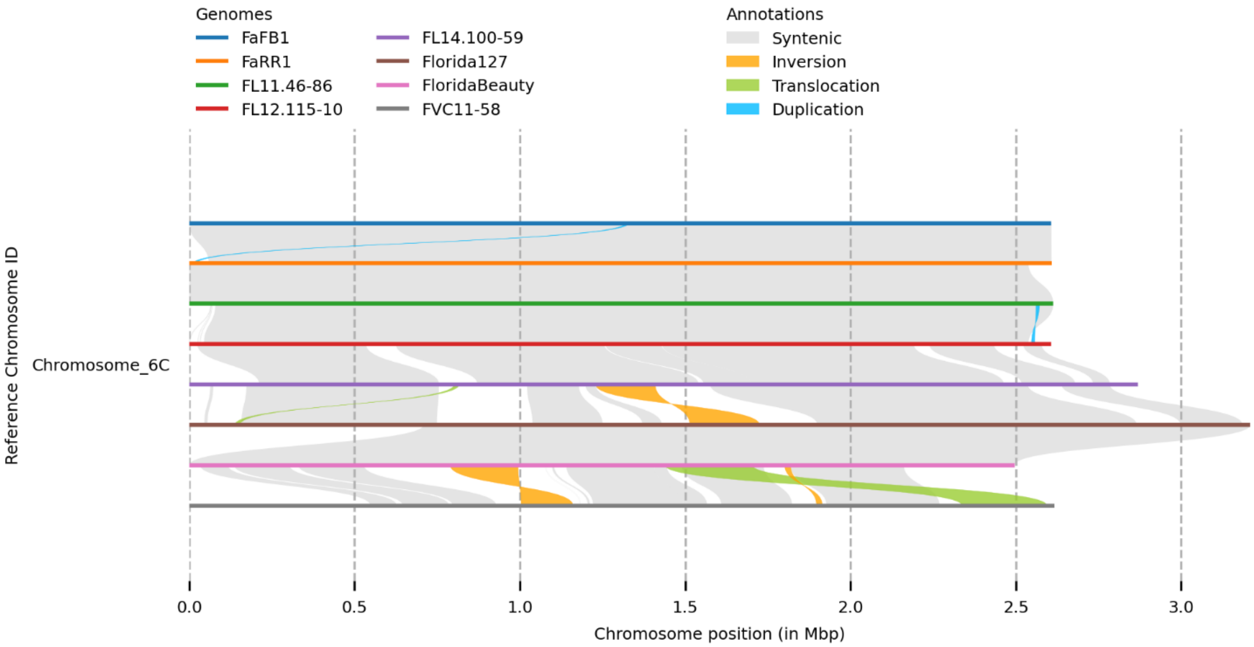

Supplementary Figure S4. Comparative genomics of eight octoploid strawberry reference genomes; ‘Florida Brilliance’ (FaFB1), Royal Royce (FaRR1), FL11.46-86, FL12.115-10, FL14.100-59, Florida127, Florida Beauty and FVC11-58.

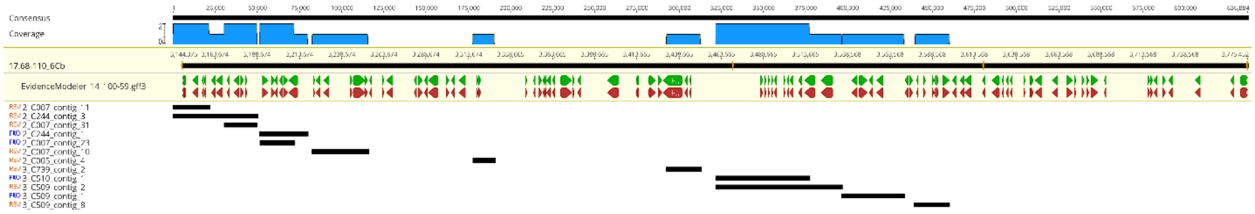

Supplementary Figure S5. Result of mapping bacterial artificial chromosome (BAC) clone contigs to a 600-kb *RXf1* in the FL14.100-59 genome.

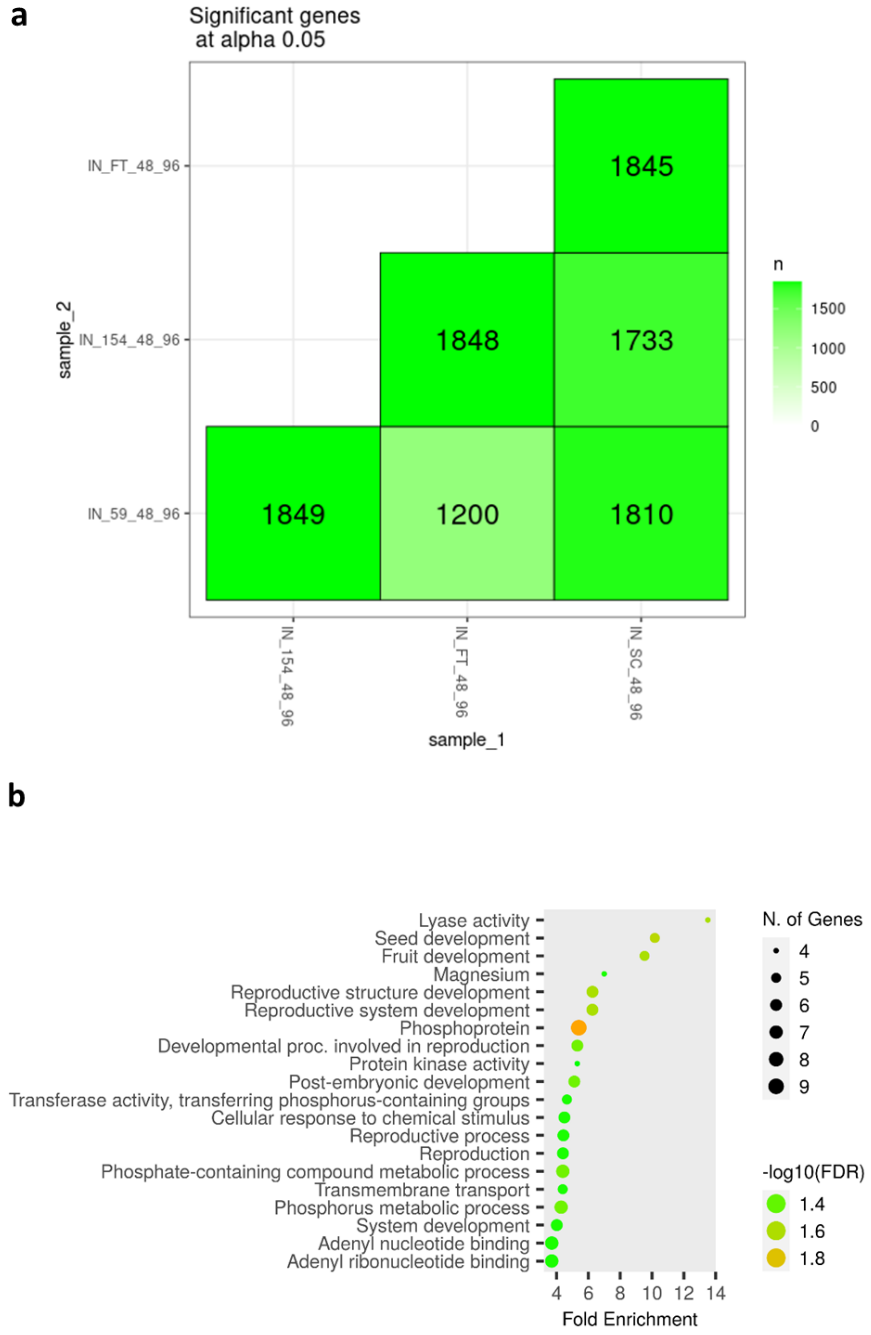

Supplementary Figure S6. **(a)** The number of significant genes from multi-directions comparison (FT: Florida Festival, 154: FL14.101-154, 59: FL14.100-59, and SC: Sweet Charlie). The label 'IN' indicates samples from *Xanthomonas fragariae* inoculation, and '48_96' indicates samples from 48 and 96 hours after inoculation combined into a group. **(b)** Dot plot of GO (Gene Ontology) of differentially expressed genes (DEGs) showing the top 20 enriched pathways.

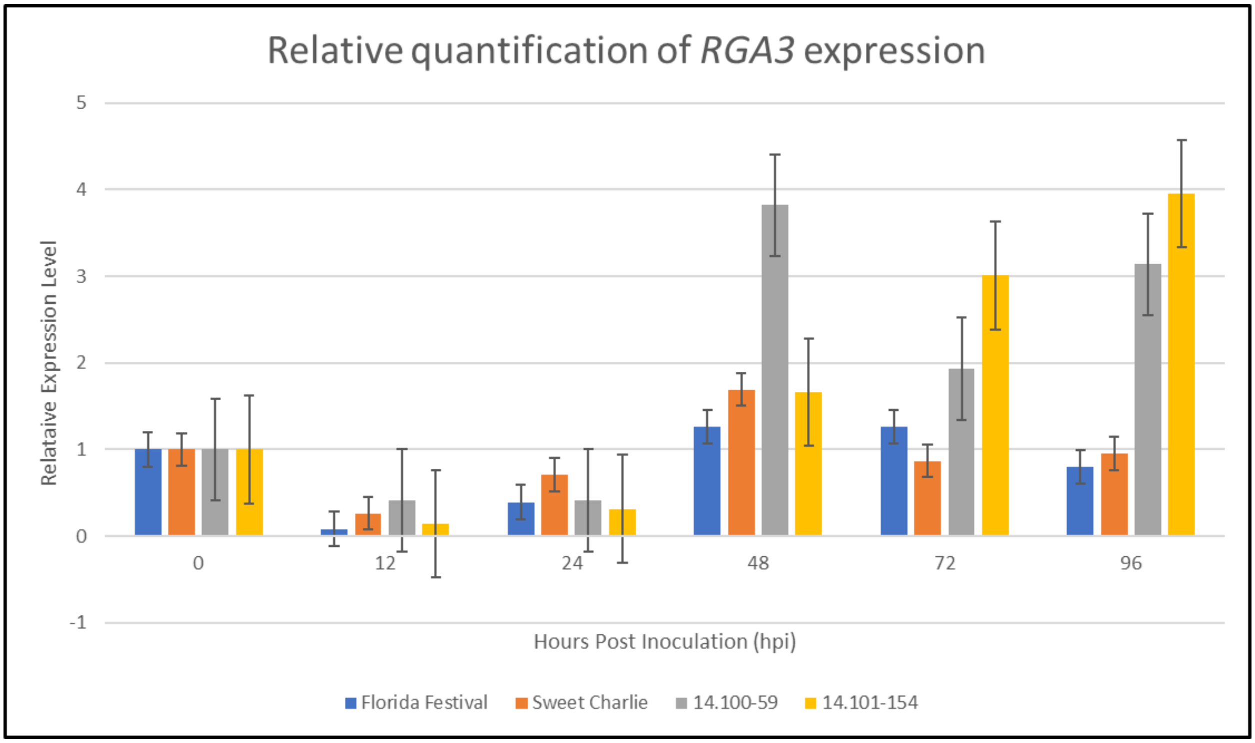

Supplementary Figure S7. Relative quantification of candidate gene *RGA3* expression in four cultivars at six different time points. Reverse transcript quantitative PCR (RT-qPCR) was conducted with the housekeeping gene *FaPDH* as a reference.

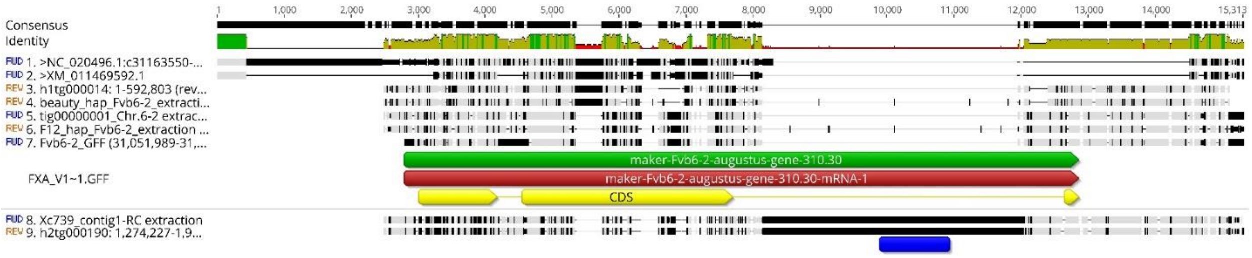

Supplementary Figure S8. Genomic sequence alignment of nine genomic sources for *RGA3* (1: genomic sequence from *Fragaria vesca*, 2: mRNA sequence from *F. vesca*, 3: genomic sequence from Florida127, 4: genomic sequence from Florida Beauty, 5: genomic sequence from FaFB1, 6: genomic sequence from FL12.115-10, 7: genomic sequence from 'Camarosa', 8: genomic sequence from FL14.101-225, 9: genomic sequence from FL14.100-59). Lane 8 and 9 are the ALS resistant genotypes. The blue annotation indicates the 3,820-bp insertion.

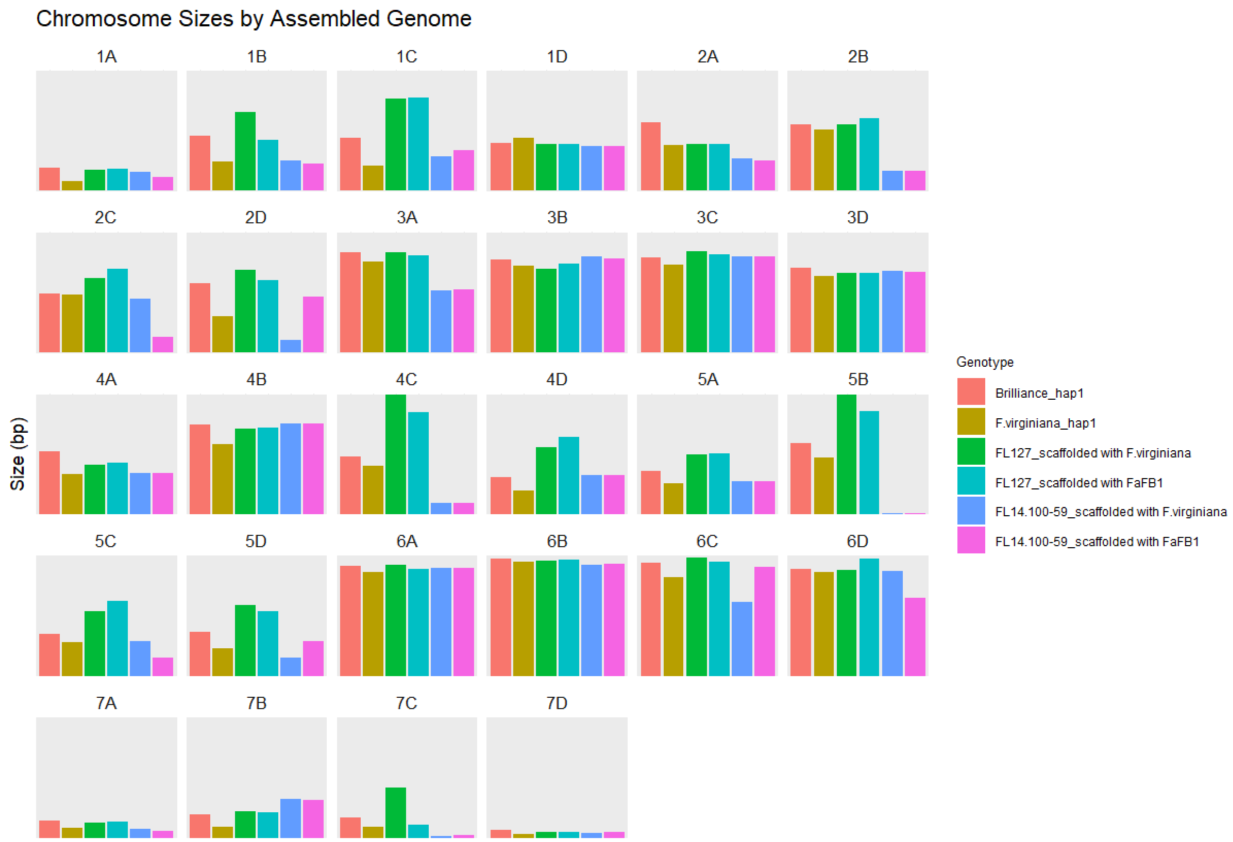

Supplementary Figure S9. Comparison of chromosome sizes between genomes. The first two blocks represent the chromosome sizes of the octoploid strawberry reference genomes, 'Florida Brilliance' (FaFB1) and *Fragaria virginiana*, respectively. The subsequent two blocks correspond to the assembled genomes of haplotype 1 ('FL127') from FL17.68-110, scaffolded using the *F. virginiana* and FaFB1 reference genomes. The final two blocks represent haplotype 2 (FL14.100-59) from FL17.68-110, scaffolded using the *F. virginiana* and FaFB1 reference genomes.

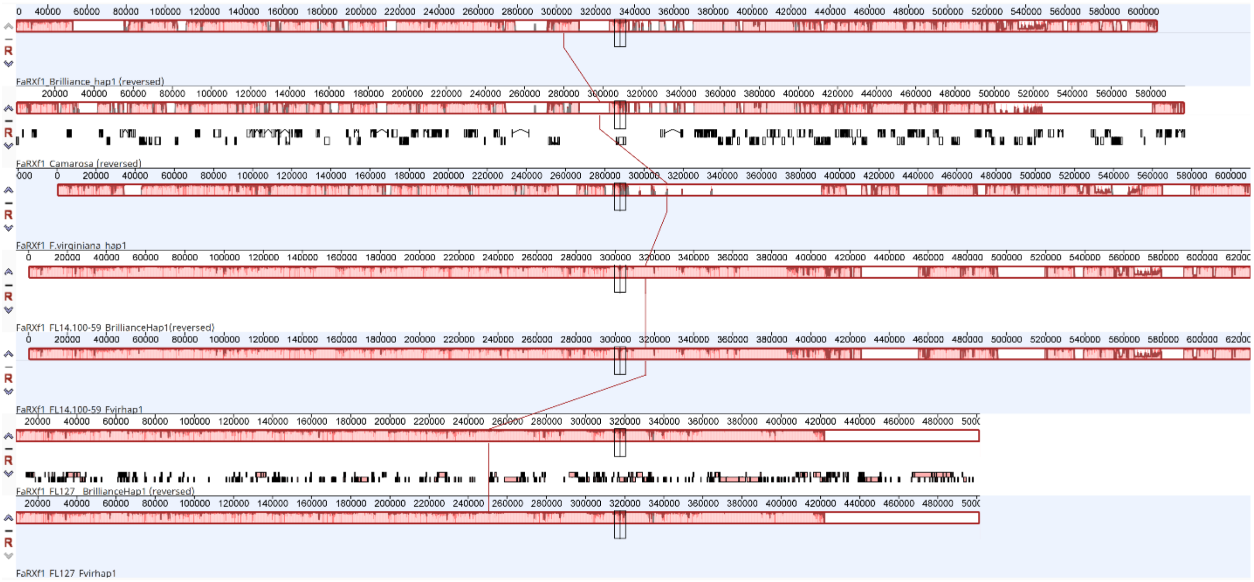

Supplementary Figure S10. Comparison of the genomic structure of *RXf1* across seven genomes. From the top lane downward, each lane represents the *RXf1* region derived from: ‘Florida Brilliance’ (FaFB1), ‘Camarosa’, *Fragaria virginiana*, FL14.100-59 scaffolded using FaFB1, FL14.100-59 scaffolded using *F. virginiana*, ‘Florida127’ scaffolded using FaFB1, and ‘Florida127’ scaffolded using *F. virginiana*.

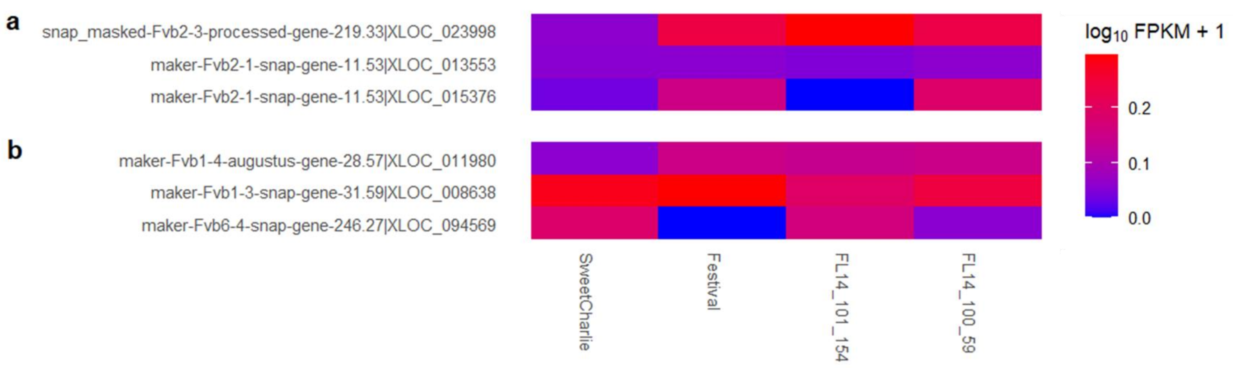

Supplementary Figure S11. The expression of *PR1* (*pathogenesis-Related 1*, a) and *FRK1* (*Flagellin Receptor Kinase 1*, b) in four cultivars, Sweet Charlie, Festival, FL14.101-154, and FL14.100-59.

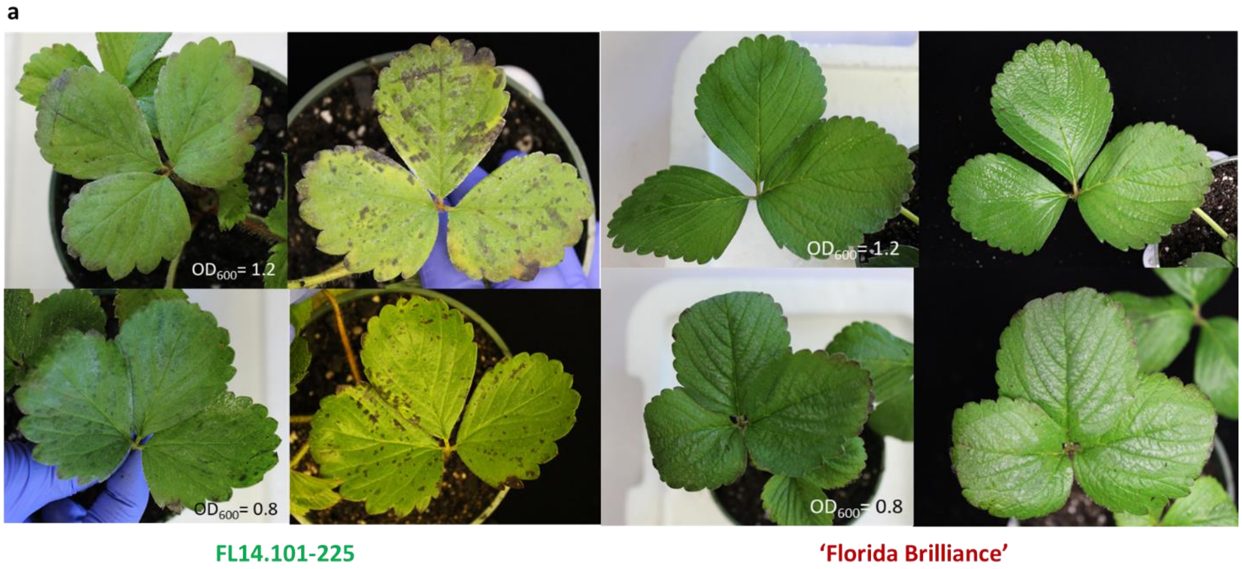

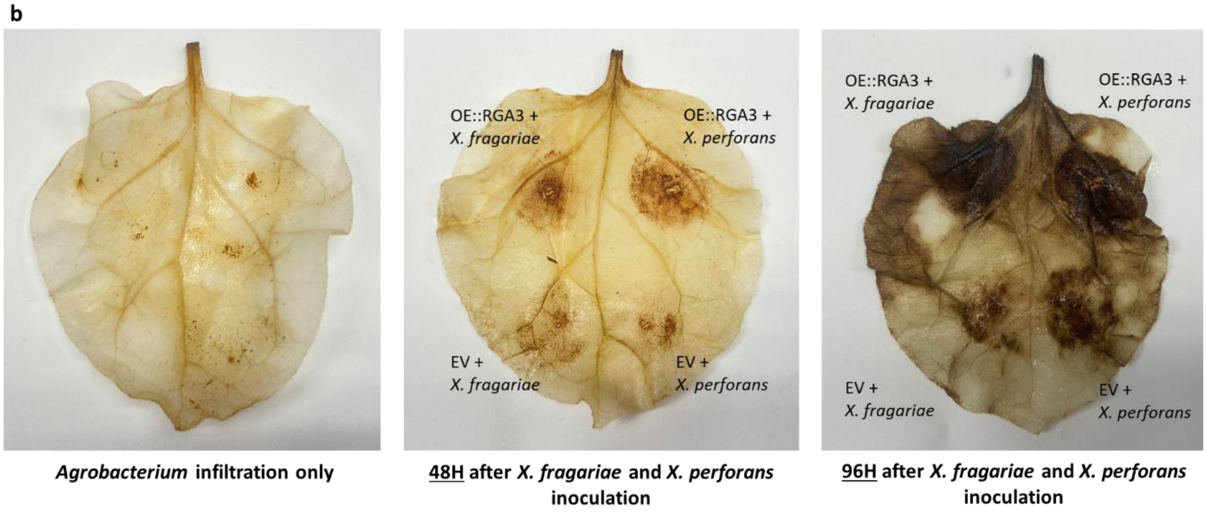

Supplementary Figure S12. Detection of hypersensitive reactions (HR) in octoploid strawberry cultivars and *Nicotiana tabacum*. **(a)** Two different concentrations of *Xanthomonas fragariae* inoculum, OD_{600} 1.2 and 0.8, was vacuum-infiltrated into two strawberry cultivars, ALS-resistant FL14.101-225 and -susceptible 'Florida Brilliance'. The HR was observed at 24 and 96 hours after inoculation. **(b)** Transient overexpression of *RGA3* with inoculation of *X. fragariae* and *X. perforans* (bacteria causing bacterial spot in tomato and pepper) was performed in *Nicotiana tabacum*. Reactive oxygen species (ROS) was observed by diaminobenzidine tetrahydrochloride (DAB) staining.

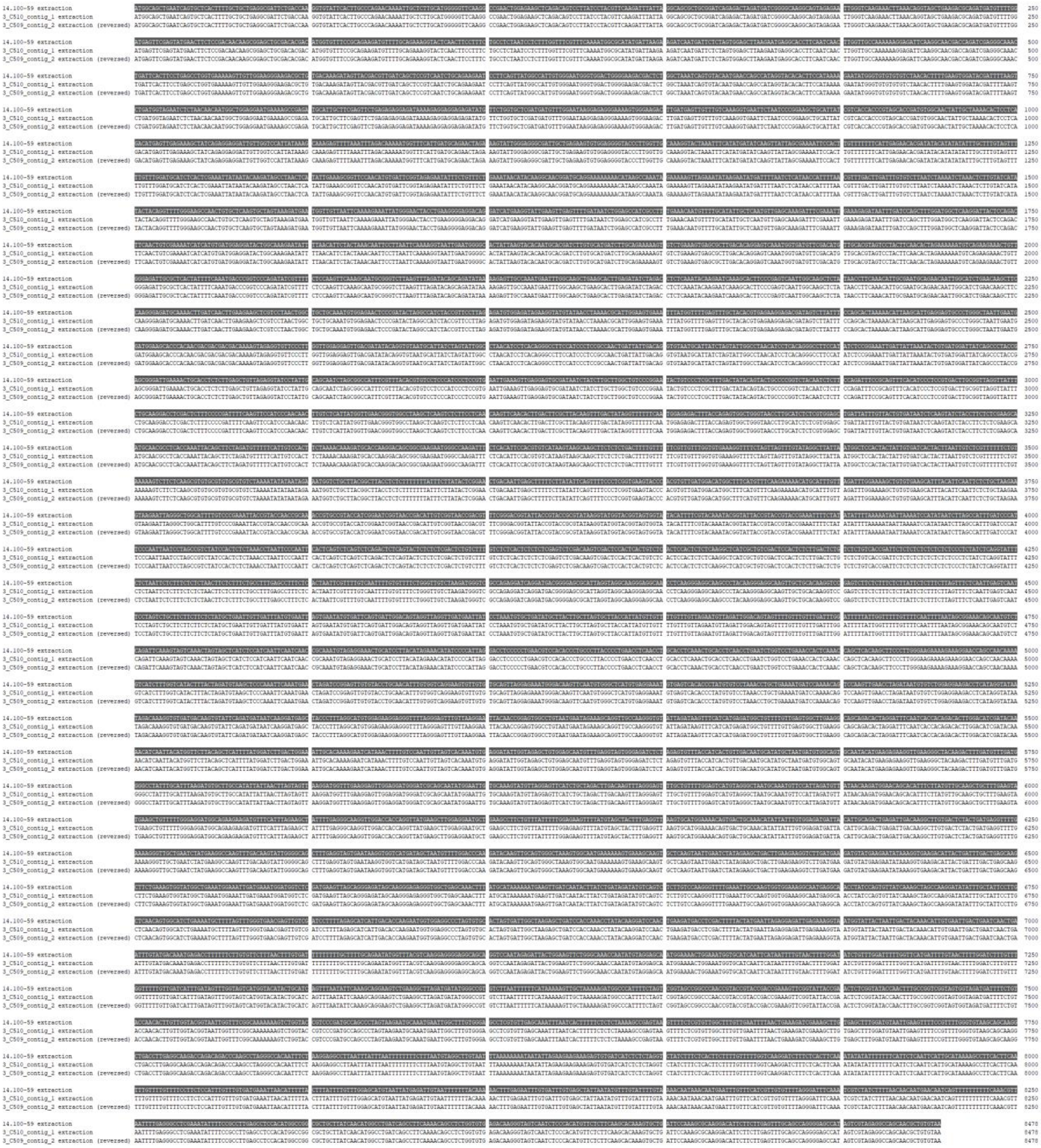

Supplementary Figure S13. Genomic sequence alignment of three genomic sources for *RGA3*.

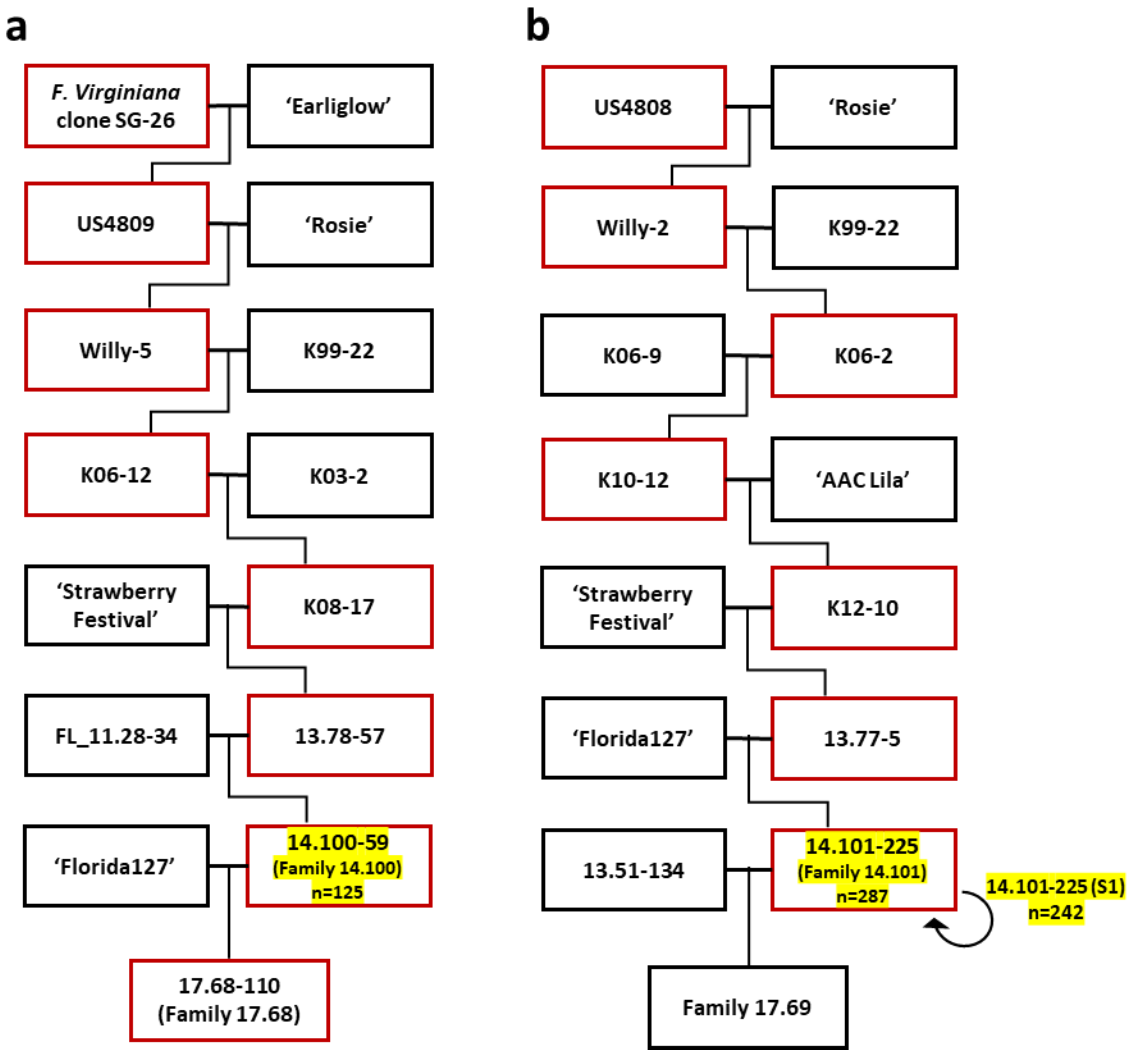

Supplementary Figure S14. Pedigree map of full-sib families used and generated in this study. (**a**) Family 14.100. (**b**) families 14.101 and selfing population 14.101-225. Red boxes indicate resistant individuals or families segregating for resistance. This figure is adapted and modified from Roach et al., 2016.

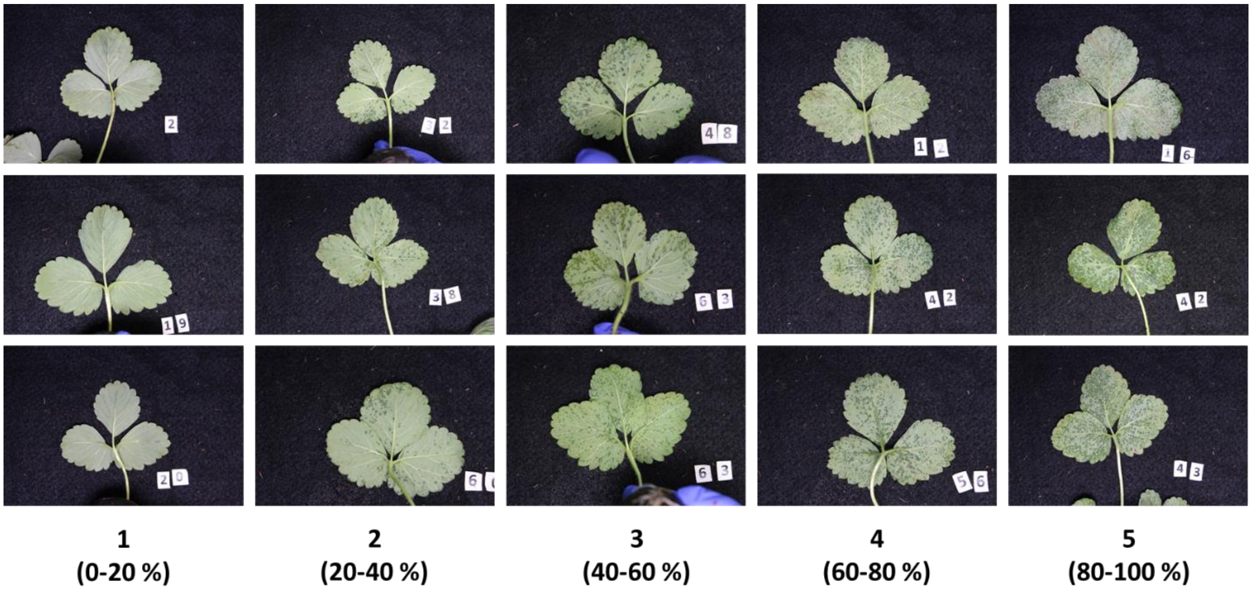

Supplementary Figure S15. Disease index for angular leaf spots in octoploid strawberry. Individual plants were scored for percent diseased leaf area on five scales from 0 to 100% with 20% increment.

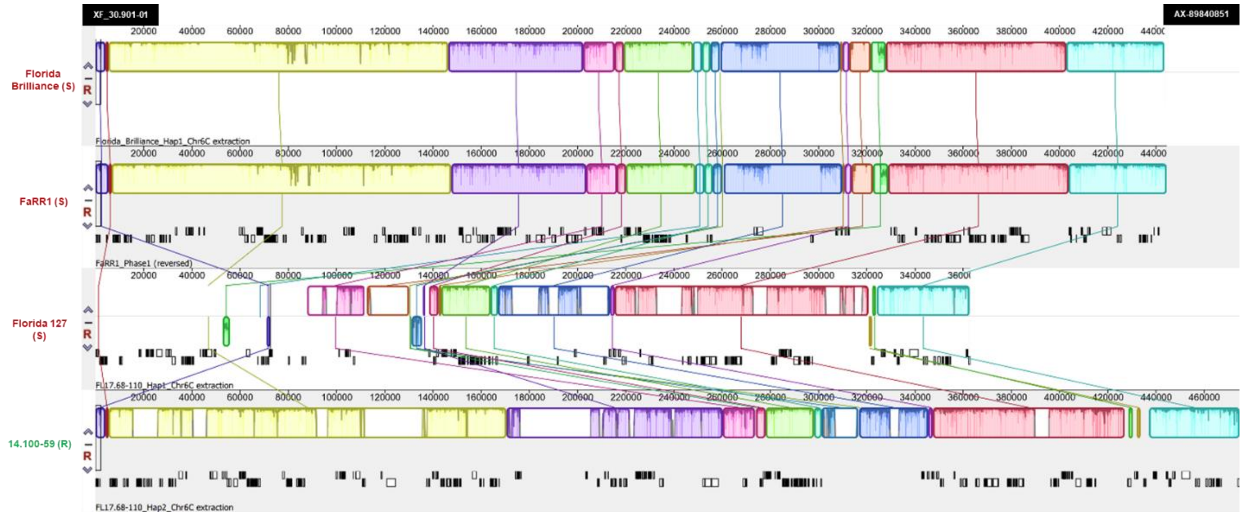

Supplementary Figure S16. Genomic structure of *FaRXf1*. The structural variations and rearrangements between four sequences, 'Florida Brilliance' (FaFB1), Royal Royce (FaRR1), 'Florida127' and FL14.100-59, were diverse in the interval region between marker XF_30.901-01 and AX-89840851.
